# Single-cell RNA sequencing identifies microglial state changes associated with iTBS after ischemia–reperfusion injury

**DOI:** 10.1371/journal.pone.0346888

**Published:** 2026-04-20

**Authors:** Yingying Huang, Yining Zhao, Xingyu Zhang, Xia Bi

**Affiliations:** 1 Graduate School of Shanghai University of Traditional Chinese Medicine, Shanghai, China; 2 Department of rehabilitation medicine, Shanghai University of Medicine and Health Sciences Affiliated Zhoupu Hospital, Shanghai, China; 3 Department of Sport Rehabilitation, Shanghai University of Sport, Shanghai, China; University of Rijeka Faculty of Health Studies: Sveuciliste u Rijeci Fakultet zdravstvenih studija, CROATIA

## Abstract

Understanding how to effectively treat neurological deficits after stroke remains difficult, as both the therapeutic targets and clinical outcomes are still uncertain. Although intermittent Theta burst stimulation (iTBS) has recently been shown to protect neural tissue in stroke-induced rats, its underlying biology has not been clearly defined. To clarify how iTBS acts at the cellular level, we applied single-cell transcriptome sequencing to a rat model of middle cerebral artery occlusion. After receiving the iTBS protocol, rats underwent behavioral assessments to determine the extent of neurological recovery. We then profiled gene expression across neuronal subtypes using 10x Genomics technology. iTBS markedly alleviated neurological impairment, and single-cell profiling indicated that the stimulation was associated with changes in microglial transcripts (e.g., *Vav3* and *Cblb*) alongside coordinated responses in other cell populations. Together, these data suggest that iTBS is associated with changes in post-ischemic microglial state composition and transcriptional programs, highlighting candidate neuroinflammatory processes for future mechanistic investigation.

## Introduction

Across the globe, countless individuals suffer disability or death when the brain’s blood flow is suddenly disrupted—a pathological event known as ischemic stroke, in which neurons perish and neurological functions deteriorate as a consequence of oxygen deprivation. The economic burden of ischemic stroke is enormous, with the costs of acute medical care, rehabilitation, and long-term disability placing a heavy strain on the healthcare system and society [1]. Current diagnostic and therapeutic strategies focus on the timely restoration of blood flow through thrombolysis and thrombectomy; the widely recognized conservative therapeutic agents are tissue plasminogen activators; however, these interventions are limited by a narrow time window (6-hour time limit for mechanical thrombolysis, and 4.5-hour time limit for pharmacological thrombolysis) and the risk of bleeding complications. As a result, only 5% of stroke patients are able to benefit from the therapeutic window to be treated [2]. Furthermore, existing treatments fail to adequately address the complex pathophysiological changes that occur after stroke, including the diverse responses of various brain cell types and the dynamic gene expression profiles associated with ischemic injury. This highlights a critical research gap, as a comprehensive understanding of the cellular and molecular mechanisms underlying ischemic stroke is essential. Essential for the development of more effective therapeutic strategies, thereby underscoring this point.

The present study focused on identifying the major brain cell types and their specific gene expression profiles in the context of ischemic stroke, a condition that has been widely investigated in previous studies [3,4]. Previous research has shown that after ischemic stroke, microglia transition into various states and infiltrate the brain tissue. Tively leading to a complex immune response [5–7]. This immune response represents a crucial therapeutic target for ischemic stroke. Notably, differentially expressed genes (DEGs) and their associated pathways have been implicated in the pathophysiology of ischemic stroke, underscoring the pivotal role of cellular responses in disease progression. Our study reveals a distinct gene expression signature in a subpopulation of microglia, emphasizing the heterogeneity and adaptive response of these immune cells to ischemic injury. Furthermore, we demonstrate that ischemic stroke significantly increases the diversity of intercellular communication within the brain, suggesting that there may be complex interactions between various cell types that may influence overall disease progression. These findings not only deepen our understanding of cellular dynamics following ischemic events, the potential for targeted therapeutic strategies to improve clinical outcomes by modulating these interactions.

In parallel with advances in cellular neuroimmunology, accumulating evidence from recent systematic reviews indicates that repetitive transcranial magnetic stimulation (rTMS), including intermittent theta-burst stimulation (iTBS), has been increasingly reported to exert anti-inflammatory and immunomodulatory effects in both experimental and clinical neurological disorders [8–10]. These effects include attenuation of pro-inflammatory cytokine release, regulation of microglial activation states, and modulation of peripheral immune–central nervous system crosstalk. However, despite growing recognition of these immunological actions, the cellular and molecular substrates through which iTBS reshapes post-stroke neuroinflammation remain incompletely defined at single-cell resolution.

## Materials and methods

### Method of euthanasia

Euthanasia by carbon dioxide is performed by placing the animal, such as a laboratory rodent, into a sealed chamber. Pre-compressed CO₂ gas is then introduced into the chamber at a controlled flow rate, typically displacing 10–30% of the chamber volume per minute. This gradual influx allows the atmospheric oxygen to be slowly replaced, causing the animal to lose consciousness due to hypercapnia (excess CO₂ in the bloodstream) before hypoxia (lack of oxygen) occurs, which minimizes distress. Once unconsciousness is achieved, the CO₂ concentration is maintained at a high level (usually above 70%) for a specified period to ensure respiratory arrest and death.

### Alleviation of suffering and humane endpoints

Throughout the study, efforts to prevent animal pain and discomfort were prioritized. Instead of waiting for severe symptoms to appear, trained staff conducted daily checks to identify any early indications of illness, distress, or pain. A set of humane endpoints—such as persistent weight loss of more than 20% from baseline, marked lethargy, pronounced infections, or an inability to reach food or water—guided decisions about intervention. Once an animal met any of these criteria, the previously specified euthanasia procedure was carried out without delay to avoid additional suffering. Conditions in the housing environment were also arranged to support species-appropriate welfare; temperature and lighting were regulated, cages were enriched, and food and water were provided freely unless an experimental requirement dictated otherwise.

### Animal

Sprague–Dawley rats, all 8-week-old males with uniform genetic backgrounds, were supplied by Animal Experiment Jihui in Shanghai. All animals were maintained in a facility free of specific pathogens. The housing environment was carefully regulated, keeping the temperature between 20 °C and 25 °C and relative humidity at 50–70%. A regular light–dark alternation every 12 hours provided a stable illumination pattern. Throughout the study, the 80% animals had unrestricted access to food and water. Experimental procedures followed the 1996 Guide for the Care and Use of Laboratory Animals issued by the National Institutes of Health and the National Research Council. Ethical approval for the study was obtained from the independent Ethics Committee of Zhoupu Hospital, Shanghai Pudong New Area (approval ID: ZPYYLL-2018-02, Shanghai, China), and all efforts were made to minimize animal suffering. Following successful establishment of the MCAO/R model, animals assigned to the MCAO group were left untreated for the remainder of the experiment. In contrast, iTBS intervention was initiated in the iTBS group 6 hours after reperfusion surgery, in accordance with a previously reported protocol [10].

For animals assigned to the iTBS group, magnetic stimulation was delivered using a CCY-I transcranial magnetic stimulator (Wuhan Yiruide, Wuhan, China) equipped with a custom-designed circular Y064 coil. The coil had an outer diameter of 64 mm, with an effective winding outer diameter of 55 mm and an inner diameter of 16 mm. The winding consisted of copper tubing (outer diameter 3 mm, inner diameter 1.8 mm), arranged in 5 turns across 6 layers. These geometric parameters fully define the electromagnetic characteristics of the stimulation coil and were used consistently in both experimental application and computational modeling.

During stimulation, the coil was positioned tangentially in direct contact with the scalp and centered over the left cortical region corresponding approximately to the primary motor cortex. Coil positioning was guided by anatomical landmarks and maintained consistently across sessions (see S1 Fig). In rodents, the distance between the scalp surface and cortical tissue is on the order of millimeters; based on established multilayer head models and μCT-derived anatomical reconstructions, this distance is typically estimated to be approximately 1.2–2.3 mm depending on anatomical location [11,12].

The stimulation protocol followed a standard intermittent theta-burst stimulation (iTBS) paradigm. Stimulation intensity was set at 120% of the resting motor threshold. Each burst consisted of three pulses at 50 Hz, repeated at 5 Hz. Stimulation was delivered in 2 s trains followed by 8 s inter-train intervals, resulting in a total of 600 pulses per session. Sessions were administered once daily for 7 consecutive days.

To characterize the spatial distribution of the induced electromagnetic fields, finite-element simulations were performed using COMSOL Multiphysics. The coil geometry, conductor properties, and stimulation parameters used in the simulations were identical to those described above. The model incorporated tissue conductivity values consistent with established rodent head models. As shown in S1 Fig, the simulations provide the distribution of magnetic and induced electric fields, as well as the decay of electric field strength with distance from the coil surface. Based on these simulations, the peak electric field at the cortical surface was estimated to fall within the range typically reported for rodent TMS studies (on the order of tens to over 100 V/m, depending on depth and orientation).

Together, these detailed specifications of coil geometry, positioning, and stimulation parameters are provided to ensure full reproducibility of the neuromodulation protocol.

This intervention was applied once daily over a continuous 7-day period. Each stimulation session consisted of theta-burst magnetic pulses delivered at an intensity corresponding to 120% of the resting motor threshold. Within each burst, stimuli were administered at 50 Hz, whereas bursts recurred at a frequency of 5 Hz [13]. The stimulation pattern involved 2 seconds of active stimulation followed by an 8-second pause, resulting in a cumulative total of 600 pulses per session. Animals in the sham-operated group underwent procedures identical to those of the iTBS group, including anesthesia induction, body fixation, coil positioning, daily handling, and adherence to the same stimulation timetable. Nevertheless, effective magnetic stimulation was intentionally avoided. To achieve this, the coil was positioned 15 cm above the rat’s head, a distance exceeding the functional range required to elicit neuromodulatory effects [13].

For single-cell RNA sequencing (scRNA-seq), brain tissue samples were collected from independent animals in the Sham (n = 2), MCAO (n = 3), and iTBS (n = 3) groups, with each animal processed as an individual biological sample. For behavioral assessments, an independent cohort was used with five animals per group (Sham, n = 5; MCAO, n = 5; iTBS, n = 5). Quantitative real-time PCR (qPCR) validation was performed using three independent biological replicates per group (n = 3).

Before experiments began, the animals were randomly allocated to three experimental conditions: a sham operation group, an MCAO group, and an iTBS-treated group (five rats in each). Behavioral assessments were performed on the same individuals that were later euthanized on day 4, at which point brain tissue was collected for RT-qPCR.

### Modeling cerebral ischemia–reperfusion via MCAO in rats

Cerebral ischemia–reperfusion injury was induced in rats using a modified middle cerebral artery occlusion (MCAO) technique as previously reported [12,13]. After overnight fasting with free access to water, rats were anesthetized with 2% isoflurane (70% N₂/30% O₂) and mechanically ventilated. Under sterile conditions, the right common, external, and internal carotid arteries were exposed. The common carotid artery was temporarily ligated, and a silicone-coated filament (0.26 mm) was inserted through the external carotid artery into the internal carotid artery to block the middle cerebral artery for 120 min, followed by reperfusion upon filament withdrawal. Sham-operated rats underwent the same procedure without arterial occlusion.

### Behavioral test

The rats were placed in the central compartment of theopen-field box (the bottom of the box consisted of 36 small squares of 16.7 cm × 16.7 cm, of which the peripheral 32 compartments were for the outer field range of motion and the center 4 compartments were for the central range of motion), and the photographic equipment was placed on the absent-field box, which was required to be able to fully observe the rat’s trajectory of movement in order to provide a complete image of the rat’s activity [9]. After 1 minute of acclimatization, the total distance traveled by the rats in the field box was recorded over a period of 5 min. Between the 2 experiments, rat feces and urine were removed, and 75% ethanol was used to remove the odor [14].

### Single-cell suspension preparation

The cells were first checked for quality and counted, ensuring a viability of at least 80%. Following this, the cells underwent washing, resuspension, and adjustment to a final concentration ranging from 700 to 1200 cells/ul. This prepared suspension was then ready for the on-board processing of the 10x Genomics Chromium™ system.

### Library construction

In a microfluidic chip, oil droplets encapsulate individual cells, necessary reagents, and Gel Beads that carry cell barcodes, resulting in the formation of Gel Beads-in-Emulsions (GEMs). Once inside these droplets, the cells undergo lysis, releasing RNA. Under optimal conditions, the RNA binds with a Poly(dT) primer that includes a cell barcode and a Unique Molecular Identifier (UMI), which facilitates the extension of the complementary strand. At this stage, three C bases are added to the extended strand’s end. The reverse strand extension is then completed using TSO as a template, where the CCC sequence pairs complementarily with the rGrGrG sequence of TSO. This process ensures that the CCC effectively matches the TSO to complete the reverse transcription. Following the reverse transcription, the GEMs are broken apart, and the Complementary DNAs (cDNAs) are isolated and amplified by PCR to create cDNA libraries.

### On-line sequencing

The completed libraries were sequenced using Illumina Hiseq or NovaSeq platforms in PE150 sequencing mode, with a typical sequencing volume of 20k reads/cell and above.

### Bioinformatic analysis

To enhance analytical robustness and minimize pipeline-specific bias, we adopted a multi-tool scRNA-seq analysis strategy. Raw sequencing data were initially processed using Cell Ranger (10x Genomics) for alignment, filtering, and gene–cell matrix generation. Downstream analyses were then performed using both Scanpy (Python-based) and Seurat (R-based) frameworks, which implement conceptually similar but independently developed algorithms for normalization, dimensionality reduction, clustering, and differential expression analysis.

The use of multiple pipelines was intended as a cross-validation strategy to ensure that major transcriptional patterns and cell-type annotations were consistent across analytical frameworks, rather than to generate independent statistical inferences. All biological interpretations presented in this study are based on concordant results observed across pipelines.

The matrix generated through Cell Ranger then served as the input for downstream analysis in Scanpy. With Scanpy, we continued the workflow by standardizing cell profiles, removing low-quality cells, grouping cells into subpopulations, and conducting differential-expression testing. Marker genes characteristic of each subgroup were subsequently identified through this analysis.

Because the number of biological replicates per group was limited, downstream differential expression and pathway enrichment analyses were interpreted as exploratory and hypothesis-generating rather than definitive inferential tests of mechanism.

### Cell identification results

In this experiment, researchers uploaded eight rat samples, from which 70,457 cells were initially identified. After applying quality-control filters, 67,120 cells remained for downstream analysis. The dataset was then subjected to clustering, producing 17 distinct groups. These clusters were subsequently assigned putative identities, revealing a range of cell populations such as astrocytes, excitatory neurons, oligodendrocyte precursor cells, mature oligodendrocytes, microglia, endothelial cells, fibroblasts, monocytes, and additional neuronal subtypes.

### Cell subpopulation identification

Instead of beginning with downstream visualization, the analysis workflow first processed the raw sequencing output with Cell Ranger, which produced the quantified gene–cell matrices used in all subsequent steps. These matrices were then examined in Scanpy, where we removed low-quality cells, normalized expression values, and corrected batch-dependent variation. After these preparatory steps had been completed, dimensionality-reduction methods—including PCA, followed by either t-SNE or UMAP—were applied to project cells into lower-dimensional spaces.

Building on these embeddings, we clustered the cells, identified cluster-specific marker genes, and ultimately assigned biological annotations to each group. Techniques such as stochastic neighbor embedding, UMAP visualization, cluster detection, marker identification, and annotation were all integrated into this analysis pipeline, though executed at different stages depending on their function.

### Cellular filtration

In single-cell transcriptome sequencing, barcodes and UMIs are assigned to gel beads to mark individual cells and their transcripts. However, a bead may occasionally enclose no cell or multiple cells, and damaged or lysed cells often release excessive mitochondrial RNA. To ensure that only intact, biologically meaningful cells proceed to downstream analysis, each cell must be evaluated using three metrics: total UMI count, mitochondrial-gene proportion, and the number of detected genes.

For this project, filtering was carried out according to the following criteria.

First, cells showing more than 20% mitochondrial transcripts were removed, since elevated mitochondrial RNA usually indicates apoptosis or membrane rupture.

Second, cells with fewer than 100 UMIs were excluded to eliminate droplets with extremely low transcript capture.

Third, we retained cells expressing 500–7000 genes, a range that helps distinguish genuine single-cell profiles from droplets containing multiple cells or from ruptured cells with abnormally low complexity.

In addition, genes with unreliable detection were also filtered out: only genes observed in at least 10 cells were considered robust. For each sample, distributions of gene counts, UMI counts (nUMI), and mitochondrial-gene percentages (percent.mt) were calculated both before and after quality control.

### Dimensionality reduction and clustering

After filtering, the expression matrix was subjected to dimensionality reduction clustering analysis using Scanpy:

1. Expression homogenization of the data was first performed to correct for sequencing depth and eliminate technical noise;
2. Highly variable genes (highly characterized genes between cells) were then selected and used to improve cell clustering accuracy (default value is 2000);
3. Multiple samples need to merge the data before subsequent analysis, you can choose whether to remove the batch effect to deal with the differences arising from samples in different batches that are not related to biocomplexity, and we use the BBKNN (Batch Balanced K Nearest Neighbours) algorithm to correct the batch effect, so as to realize the integration of different single-cell datasets;
4. After further homogenization of the expression values, the data were downscaled using PCA analysis, which is a linear downscaling method that applies variance decomposition to map the high-dimensional data into a low-dimensional space; the cells were then clustered and subclustered based on the SNN clustering algorithm to construct the clustering relationship between cells;
5. Finally, the dimensionality-reduced data are passed to t-SNE and UMAP for visualization and display, the more similar the gene expression patterns between cells are, the closer the distance is in the t-SNE/UMAP graph.

### Visualization of clustering results

After the dimensionality reduction is completed, we use a graph-based clustering method to partition this graph into highly correlated categories by embedding cells into the graph and then drawing edges between cells with similar expressions using the K-nearest neighbor method. We use UMAP to visualize the results of clustering, the core idea of UMAP is to measure the similarity of points in high-dimensional space and then sort the data in low-dimensional space. However, UMAP constructs a high-dimensional map of the data and then preserves the similarity in the high-dimensional space as much as possible in the low-dimensional map representation. UMAP can separate groups from each other more clearly than tSNE. UMAP plot of all clusters, different colors indicate different clusters.

### Characterization of genes

After the cells were grouped through the clustering procedure, each group was examined for patterns of gene activity. Instead of starting from predefined marker lists, we inferred them by comparing the expression profile of one cluster against all remaining cells. To accomplish this, Scanpy’s Wilcoxon rank-sum test—implemented as a nonparametric method for differential expression—was applied to cluster 0 first and then iteratively to the other clusters. This approach allowed us to pinpoint genes that showed uniquely elevated or reduced expression within each group. Only genes meeting the criteria of logFC > 1 and FDR < 0.05 were retained, ensuring that the final set represented robustly and significantly enriched markers.

### Annotation of cellular subpopulations

Through differential gene analysis and Marker gene identification, we can determine the true cell type in each cell population. Currently, there are two main methods for cell type identification: one is based on existing cell type Marker genes, and the cell type corresponding to these specific Markers is identified by software or humans, i.e., mainly supervised and semi-supervised; the other is based on the existing expression profiling reference dataset, and the cell type is identified based on the similarity by the unsupervised method. These two methods have their advantages and disadvantages. The former has more human interference and may rely too much on the existing Marker. The latter approach, based on the reference dataset, can effectively avoid human interference, but is also limited by the existing data in the reference dataset, and can only identify the cell types up to a broad category. The current empirical approach is to combine the two methods for cell type identification to make the cell taxon identification results more reliable. (Based on PanglaoDB database).

### Functional enrichment analysis

To interpret the functions of the identified DEGs, we first mapped them to entries in the Kyoto Encyclopedia of Genes and Genomes (KEGG). Instead of beginning with the pathways directly, the analysis proceeded by applying a hypergeometric test—implemented through the *hyper()* function in R—to determine which KEGG pathways were significantly enriched [15].

### Post-processing and analysis of single-cell RNA-seq data

Expression matrices generated by Cell Ranger were subsequently processed in R (v3.5.0). The analysis pipeline was implemented mainly with the Seurat package (v3.1.5). Quality control filtering retained only cells with 400–4500 detected features, over 600 UMIs, and mitochondrial gene content below 10%. Integration anchors were computed from the first 20 dimensions and applied to merge datasets. After normalization and scaling using Seurat’s default settings, dimensionality reduction was achieved through principal component analysis (PCA), and the top 30 components were selected for downstream use.

Unsupervised clustering relied on a shared nearest neighbor (SNN) approach with a resolution parameter of 1, and the t-distributed stochastic neighbor embedding (t-SNE) algorithm was used for visualization. Cluster-specific markers were detected via the “FindAllMarkers” function (log2FC > 0.25; min.pct > 0.1). Differential gene expression between microglial clusters under distinct conditions was further examined with both SCDE (v1.99.1) and the Seurat “FindMarker” function.

### RNA isolation and quantitative real-time PCR (qRT-PCR)

Following a three-day iTBS intervention, rats were sacrificed, and microglial cells were purified from white matter using magnetic-activated cell sorting (MACS). Total RNA was isolated from the collected cells with TRIzol reagent (G3013, Servicebio, Wuhan, China). The purity and concentration of RNA were measured on a NanoDrop 2000 spectrophotometer (Thermo Fisher Scientific, MA, USA, RRID: SCR_018042). Using the PrimeScript™ RT reagent kit with gDNA Eraser (Takara Biotechnology, Dalian, China), 1 μg of RNA was reverse transcribed into complementary DNA according to the manufacturer’s protocol.

Quantitative PCR was then performed on a CFX Connect real-time PCR system (Bio-Rad, Hercules, USA, RRID: SCR_018064) using TB Green Premix Ex Taq™ II (Takara Biotechnology) and gene-specific primers synthesized by Sangon Biotech (Shanghai, China). The rat primer sequences are listed in Table 1. Relative expression levels of *Vav3* and *Cblb* were normalized to *β-actin*, and the fold changes in mRNA expression were computed using the 2^ − ΔΔCt method. Each group consisted of three rats, and reactions for each sample were conducted in triplicate.

**Table 1:** Primer sequences.

Target gene	Primer sequence	
*Cblb*	forward primer	GAGCTGTCTGCACGAAAGGA
	reverse primer	CGACTGCTCCTCGTACATCC
*Vav3*	forward prime	CTTGCCAACCCTGGTATGCT
	reverse primer	GGACTTAGCAAGCTGTTGCC
*β-Actin*	forward prime	GGACTTAGCAAGCTGTTGCC
	reverse primer	CACAGCTTCTCTTTGATGTCAC

### Statistical analysis

All statistical analyses for behavioral and qPCR data were performed using GraphPad Prism version 10 (GraphPad Software, USA). Data are presented as mean ± SEM. For these animal-level experiments, each data point represents one independent animal. Normality was assessed before parametric testing, and one-way ANOVA or nonparametric alternatives were applied as appropriate, followed by post hoc multiple-comparison correction when applicable.

For scRNA-seq analyses, animals rather than individual cells were considered the biological replicates. Single-cell visualizations, cell composition plots, violin plots, and cluster-distribution figures are presented primarily for descriptive purposes. Differential expression and pathway enrichment analyses derived from the scRNA-seq dataset were interpreted as exploratory and hypothesis-generating because of the limited number of biological replicates (Sham n = 2; MCAO n = 3; iTBS n = 3). Cells were not treated as independent biological replicates for formal statistical inference.

## Results

### iTBS on motor function in stroke rats

**Fig 1:**
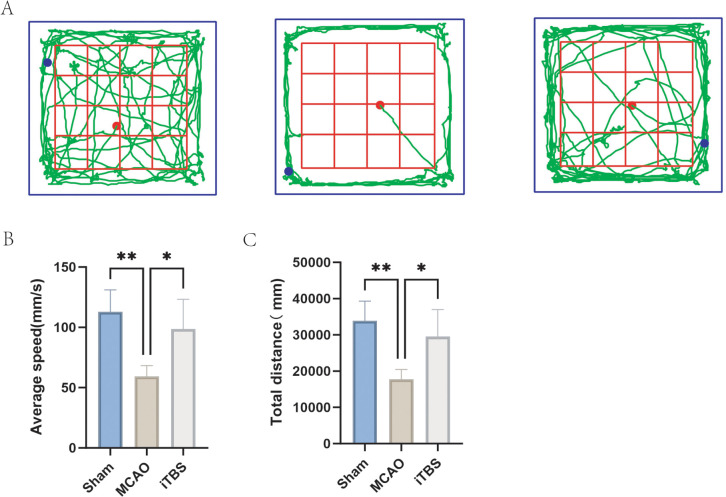
Effects of iTBS on motor function in rats after ischemic stroke. **(A)** Representative movement trajectories of rats in the open-field test. **(B)** Total distance traveled by rats in each group. **(C)** Mean movement speed of rats in each group. Data are presented as mean ± SEM. Each data point represents one independent rat (n = 5 animals per group). Statistical significance was determined using one-way ANOVA followed by appropriate post hoc tests, as described in the Methods section. *P < 0.05; **P < 0.01.

The open-field test is a common experimental method for assessing the locomotor activity of animals. The movement trajectories of the rats are shown in Fig 1A. Behavioral experiments showed that the total distance traveled by rats in the cerebral ischemia/reperfusion model induced by the wire blood clot method during the 5-minute open-field test averaged 1,770.4 mm, while rats treated with iTBS showed significantly increased locomotion, traveling 29,570.5 mm (P < 0.05) (Fig 1C). The average movement speed was 59.234 mm/s in the model group and 98.568 mm/s in the treatment group, showing statistically significant differences (P < 0.05) (Fig 1B), confirming the therapeutic effect of iTBS on stroke rats.

### Classification of predominant brain cells and identification of key genes

**Fig 2:**
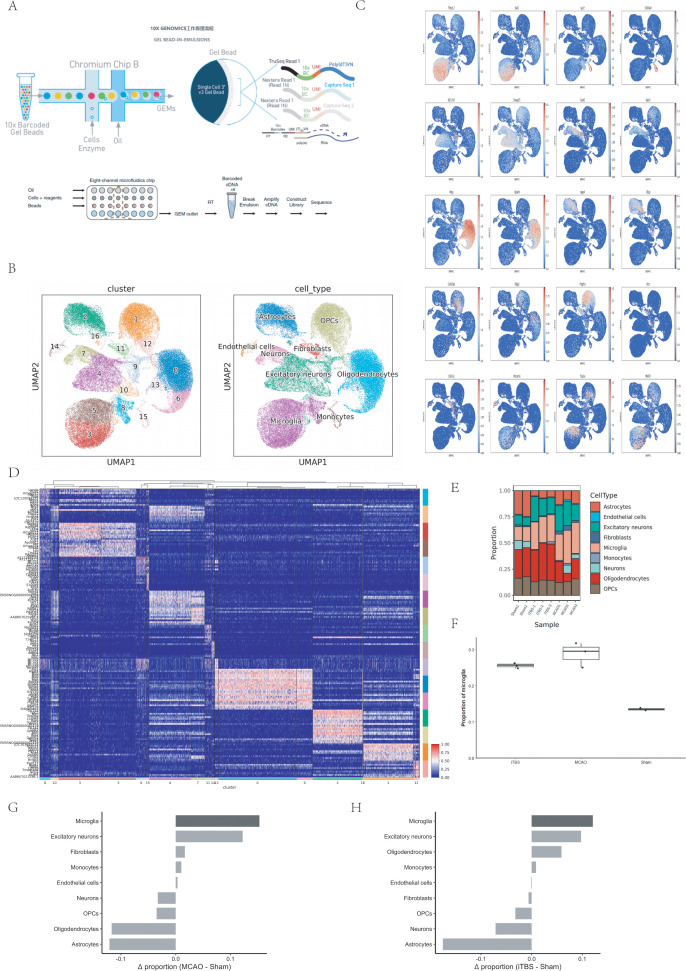
Single-cell RNA sequencing reveals cellular composition of rat brain after ischemic stroke and iTBS treatment. **(A)** Schematic overview of the single-cell RNA sequencing workflow. **(B)** t-SNE plot showing clustering of single cells colored by cell type. **(C)** t-SNE plots illustrating the expression patterns of representative marker genes across major cell types. **(D)** Bar plot showing the proportion of each cell type in individual samples. **(E)** Heatmap of the top expressed marker genes for each identified cell cluster. Each dot represents a single cell. No statistical comparisons were performed at the single-cell level. Animal-level biological replication is described in the Methods section. These compositional comparisons are shown descriptively because of the limited number of biological replicates.

Brain tissues were obtained from specific cortical regions of experimental rats under different conditions. From the sham-stimulation group—animals showing no neurological damage—the left cortex on the non-lesioned side was isolated. In contrast, cortical samples from the infarcted left hemisphere were collected from two MCAO/R model rats, together with tissues from three animals receiving iTBS treatment. To characterize how diverse cell types respond transcriptionally after stroke, single-cell suspensions were prepared and analyzed using the 10x Genomics microfluidic platform. This technology performs rapid cell encapsulation and molecular barcoding, followed by sequencing and computational profiling. Through these steps, gene expression dynamics were captured at the resolution of individual cells, enabling a detailed exploration and large-scale mapping of cellular heterogeneity in the post-stroke cortex (Fig 2A). Given the limited number of biological replicates, the single-cell dataset was used primarily to characterize cellular composition and transcriptional patterns at a descriptive level.

In this experiment, a total of 8 rat samples were processed. Following data analysis, we initially obtained 70,457 cells. After stringent quality control and cell filtration (S2 and S3 Figs), we retained 67,120 high-quality cells. These cells were derived from various sources and, upon cluster analysis, segregated into 17 distinct clusters. Preliminary annotation identified multiple cell types, including astrocytes, excitatory neurons, oligodendrocyte precursor cells (OPCs), oligodendrocytes, microglia, endothelial cells, fibroblasts, and monocytes (Fig 2B).

As shown in Fig 2C, the cell populations aggregated in this study were validated using well-established marker genes. A heatmap displaying the top 5 characteristic genes for each subfamily (Fig 2D) confirmed their identities: microglia (subfamilies 3, 5, and 8) exhibited high expression of *P2ry12* and *Sall1*; monocytes expressed *Lyz2* and *S100a9*; excitatory neurons were marked by *Slc17a7* and *Snap25*; inhibitory neurons expressed *Gad1* and *Gad2;* oligodendrocytes were characterized by *Mog* and *Opalin*; astrocytes (subfamily 2) showed expression of *Aqp4* and *Gfap*; and OPCs (subfamilies 2, 12) displayed high expression of *Pdgfra*.

Additionally, we visualized the top 3 characteristic genes for each cell type using bubble plots (S3A Fig), while violin plots further illustrated the expression patterns of the top 5 marker genes for each subpopulation (S3B Fig).

### Microglia exhibit the most pronounced and consistent compositional changes across experimental conditions

Quantitative analysis of cell-type composition revealed that multiple neural and non-neural cell populations exhibited measurable proportional changes across Sham, MCAO, and iTBS groups (Fig 2E). These alterations indicate that ischemia–reperfusion injury and subsequent iTBS intervention broadly reshape the cellular landscape of the brain.

Importantly, systematic comparison of compositional changes across all annotated cell types suggested that microglia exhibited the largest absolute proportional shift relative to Sham controls (Fig 2F). In the MCAO group, the proportion of microglia increased markedly compared with Sham animals, representing the most pronounced change among all major cell populations. Following iTBS treatment, microglial abundance was partially normalized yet remained among the cell types showing the strongest deviation from Sham levels. Ranking analyses of compositional changes further confirmed that microglia consistently occupied the top position across experimental comparisons, suggesting that microglia were among the most responsive cell populations under ischemic injury and after iTBS intervention (Fig 2G).

In contrast, astrocytes, oligodendrocyte lineage cells, neurons, and vascular-associated cells also displayed detectable compositional alterations; however, these changes were smaller in magnitude and, in several cases, exhibited context-dependent or bidirectional patterns (Fig 2D, 2F). Given the limited number of biological replicates, the present study does not aim to draw definitive functional conclusions regarding these more modest shifts. Instead, these observations are interpreted as descriptive and hypothesis-generating.

Based on the combination of (i) the magnitude of compositional change, (ii) consistency across experimental conditions, and (iii) the established role of microglia as the primary immune-responsive cells in the central nervous system, we therefore prioritized microglia for in-depth downstream analyses. Moreover, because the present scRNA-seq dataset was primarily powered to resolve immune cell–associated transcriptional programs, subsequent analyses focused on cell populations exhibiting both robust compositional changes and sufficient transcriptional signal-to-noise ratios (Fig 2H). Other cell types were therefore not subjected to equally detailed subclustering or pathway analyses.

### Unique gene expression signatures of microglia subclusters

Cerebral infarction involves a complex interplay between neuroinflammation and repair, largely governed by how microglia polarize between their M1 and M2 phenotypes [16]. Earlier investigations have shown that this phenotypic switch dictates whether microglia aggravate injury or facilitate recovery.

Cerebral infarction involves a complex interplay between neuroinflammation and repair, and microglia contribute through diverse, context-dependent activation programs. Although microglial responses have historically been described using the M1/M2 polarization framework, accumulating single-cell evidence indicates that post-ischemic microglia occupy a continuum of heterogeneous and dynamic states that cannot be captured by binary labels. Therefore, in this study we prioritize state- and program-level descriptions (e.g., immune activation/antigen presentation, complement signaling, metabolic adaptation, phagocytosis, and tissue repair) when interpreting microglial subclusters.

Evidence from experimental studies suggests that the effects of iTBS are more appropriately interpreted as reshaping the distribution of microglial functional programs toward reparative and inflammation-resolving states, rather than inducing a discrete or terminal M2 phenotype. When rats subjected to cerebral ischemia received iTBS, neuronal apoptosis was markedly reduced, and the brain’s microenvironment became more conducive to repair. These effects were accompanied by smaller infarcted regions, better neuronal survival, and preservation of synaptic structures. Moreover, iTBS appeared to dampen astrocytic and M1 microglial activation while enhancing microglial transcriptional programs associated with tissue repair and inflammation resolution [17].

Additionally, evidence suggests that modulating intracellular ROS levels can influence microglial phenotypic polarization [18]. iTBS may facilitate the shift toward the M2 phenotype by targeting relevant signaling pathways and regulating intracellular ROS levels, though the precise mechanism requires further investigation.

**Fig 3:**
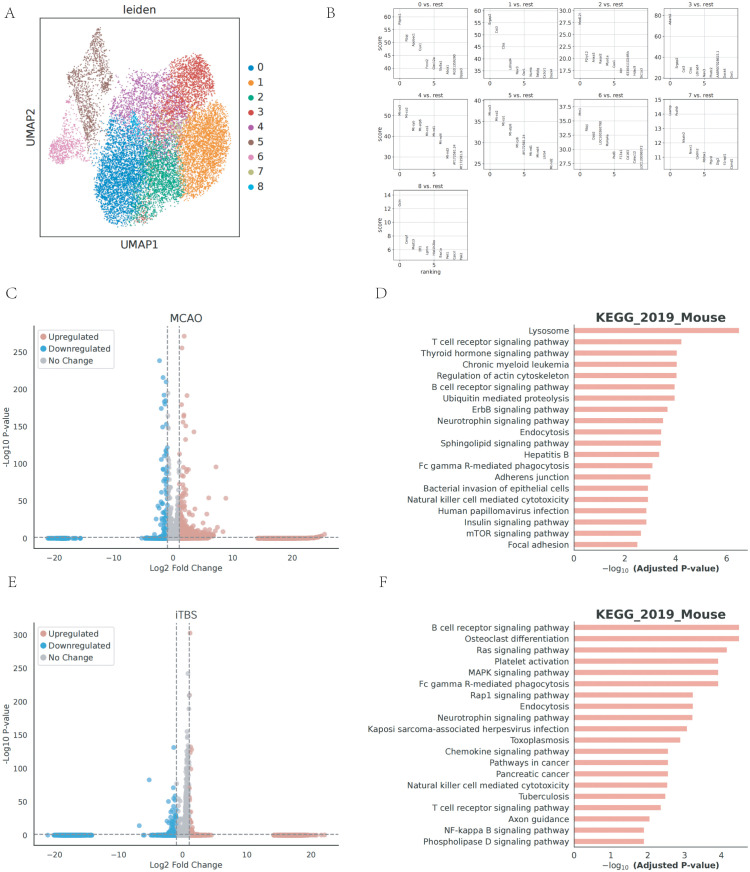
Single-cell transcriptomic analysis of microglial subpopulations. **(A)** UMAP visualization of microglial subclusters. **(B)** Heatmap showing the top marker genes for each microglial subcluster. **(C)** Volcano plot of differentially expressed genes in microglia from the MCAO group compared with the Sham group. **(D)** Functional enrichment analysis of differentially expressed genes in microglia from the MCAO group. **(E)** Volcano plot of differentially expressed genes in microglia from the iTBS group compared with the MCAO group. **(F)** Functional enrichment analysis of differentially expressed genes in microglia from the iTBS group.Differential expression analyses were conducted on aggregated single-cell transcriptomic data as described in the Methods section. Cells were not treated as independent biological replicates for statistical inference.

To gain deeper insights into microglia heterogeneity, we reclustered microglial subpopulations into 9 distinct clusters using marker genes identified by the Seurat R package [19] (Fig 3A). The MG0 cluster expresses characteristic microglial genes including *Pitpnc1* (which participates in non-vesicular lipid transport and may contribute to cellular metabolism or membrane maintenance), and *Rbpj* (a key mediator of the Notch signaling pathway that influences Cell differentiation, and *Apobec1* function as an RNA editing enzyme, which may affect inflammatory responses or cellular stress responses [20]. Taken together, the transcriptional features of MG0 may be consistent with a microglial state associated with RNA editing and metabolic homeostasis, although this interpretation remains inferential. MG1 highly expresses microglia genes: *Srgap2* (involved in cytoskeletal reorganization), *Cst3* and *Ctss. Srgap2* mediates cytoskeletal reorganization, potentially influencing cell migration or morphological changes. *Cst3* (Cystatin C) is a cysteine protease inhibitor. Inhibitor and may be involved in anti-inflammatory or neuroprotective processes. *Ctss* (Cathepsin S) is a lysosomal protease involved in antigen presentation and extracellular matrix degradation, potentially playing roles in inflammatory responses or antigen processing. These gene expressions may indicate activated microglial states, such as pro-inflammatory phenotypes, participating in antigen presentation, or tissue remodeling. MG2 expresses characteristic microglial genes: *Med12L*, *MED12L* (MediatorComplex Subunit 12L), belongs to the mediator complex, regulates transcription, and may affect cell differentiation or inflammatory responses. May be involved in the regulation of specific signaling pathways. It potentially modulates specific signaling pathways. MG2 may be involved in a regulatory subgroup of specific signaling pathways (e.g., TNF-α, IL-6 pathway). MG3 expresses microglial marker genes including *Adarb2* and MG4 expresses known microglial genes: *Mt-co3, Mt-co2*. In microglial subtype MG5, a strong transcriptional signal is observed for genes such as *Mt-co1*, *Mt-co2*, and *Mt-co3*, all of which encode subunits of cytochrome c oxidase—the mitochondrial enzyme essential for oxidative phosphorylation and ATP generation. The transcriptional profile of MG5 is suggestive of increased mitochondrial activity and may reflect a metabolically active microglial state, potentially associated with tissue repair-related programs. Similarly, MG4 also exhibits expression of *Mt-co2* and *Mt-co3*, yet lacks *Mt-co1*, indicating a slightly lower energy-driven profile compared with MG5. As both MG4 and MG5 share genes involved in the cytochrome c oxidase complex, they likely correspond to metabolically active microglial states, though MG5 may reflect a stage of intensified mitochondrial engagement associated with cellular recovery [21]. MG6 expresses known microglia genes: *Mrc1* and *Rbpj*. Although *Mrc1* is commonly referred to as an M2-associated marker, its elevated expression in MG6 should not be interpreted as evidence of a discrete M2 phenotype. Instead, the presence of Mrc1, together with immune-regulatory and metabolic genes, suggests that MG6 represents a composite microglial state enriched for tissue-repair–associated and inflammation-modulating transcriptional programs. Based on these findings, MG6 may represent a transcriptional state enriched for immune signaling- and antigen-presentation–related programs; however, this interpretation should be regarded as descriptive and hypothesis-generating. MG7 expresses known microglia genes: *Lsamp* and *Pcdh9*. *Lsamp* (Limbic System Associated Membrane Protein) is involved in cellular adhesion and neural circuit formation and may play a role in synaptic pruning or neuroplasticity [22]. *Pcdh9* (Protocadherin 9) belongs to the family of calmodulin proteins cadherin superfamily and is involved in intercellular adhesion and signaling, which may influence synaptic stability or cellular interactions. It is possible that MG7 is involved in neurodevelopment or synaptic regulation. MG8 expresses the known microglia gene *Ocln*. As Occludin (*Ocln*) is a tight junction protein that is normally expressed in epithelial cells and maintains barrier function, its expression in MG8 may be involved in blood-brain barrier maintenance or repair, or in forming connections with other cells (Fig 3B).

### Integrated transcriptomic and pathway analysis reveals microglial modulation during ischemia-reperfusion injury and iTBS intervention

To explore the heterogeneity of gene expression in differently treated sets, we investigated the pathways and biological processes involved in DEGs regulated by cerebral ischemia-reperfusion and iTBS. We summarized and compared the pathways and processes enriched for DEGs in different cell sets. We likewise found that the cells of microglia ranked high among all cells in terms of differentially expressed genes. A comparison of cell-type proportions revealed clear differences between the MCAO and sham-operated groups. In microglial cells from the MCAO group, gene expression analysis identified 13,394 genes showing increased expression and 6,654 showing decreased expression relative to the sham controls (P < 0.05) (Fig 3C). These differentially expressed genes were subsequently found to be significantly enriched across multiple biological pathways and functional processes (Fig 3D). After analysis, the main pathways enriched for the regulation of the brain by ischemia-reperfusion were as follows: Lysosome – Lysosome is the core of the intracellular degradation system and is closely related to phagocytosis of microglia, especially playing a key role in the removal of cellular debris, pathogens, or aberrant proteins (e.g., amyloid in neurodegenerative diseases); Lysosome is the core of the intracellular degradation system. Endocytosis and Fc gamma R-mediated phagocytosis – directly associated with immunophagocytosis of microglia, removing pathogens or apoptotic cells by endocytosis or antibody-dependent phagocytosis. Pathogens or apoptotic cells; mTOR signaling pathway – regulates cellular metabolism, autophagy, and inflammatory responses, and may be involved in the transition of microglia from resting to activated states or associated with neuroinflammatory regulation. Neurotrophin signaling pathway – involved in neuronal survival and synaptic plasticity, suggesting that microglia may support neuronal health or participate in neural repair through this pathway. Ubiquitin-mediated proteolysis – is associated with the labeling and degradation of aberrant proteins, and may play a role in the clearance of misfolded proteins (e.g., tau proteins in Alzheimer’s disease) by microglia. These enrichment results suggest that microglial transcriptional changes are associated with lysosomal, phagocytic, protein degradation, and inflammatory regulatory pathways, consistent with their central role in CNS immunosurveillance and homeostasis maintenance. After iTBS regulation, among them, there were 11,178 up-regulated genes and 8,873 down-regulated (P < 0.05) (Fig 3E).

KEGG pathway enrichment analysis indicated that differentially expressed genes in microglia were statistically over-represented in pathways related to immune signaling, phagocytosis, metabolic regulation, and neurotrophic support. These enrichment results reflect associations between transcriptional changes and known biological pathways, rather than direct evidence of pathway activation or inhibition. Given the limited number of biological replicates, these findings should be interpreted as hypothesis-generating and serve to highlight candidate molecular processes potentially involved in iTBS-associated modulation of post-ischemic neuroinflammation (Fig 3F). The enrichment patterns raise the possibility that iTBS is associated with shifts in transcriptional programs related to inflammatory signaling, phagocytic/endocytic processes, and immune communication. However, these pathway-level observations do not establish direct mechanisms and should be interpreted as candidate processes for future validation. DEGs are primarily associated with several key signaling pathways. These include the MAPK pathway, the NF-kappa B pathway, and the pathway involving Fc gamma receptors that mediate phagocytosis. Additionally, the DEGs are implicated in processes like endocytosis, neurotrophin signaling, and chemokine signaling.

While pathway enrichment analyses reveal significant associations with biological processes such as immune regulation and neuronal repair, these findings should be interpreted as hypothesis-generating. The observed changes in gene expression do not imply a mechanistic conclusion but rather highlight potential molecular pathways that warrant further validation through experimental studies.

### iTBS reshapes the distribution and functional transcriptional programs of microglial subclusters after ischemic stroke

**Fig 4:**
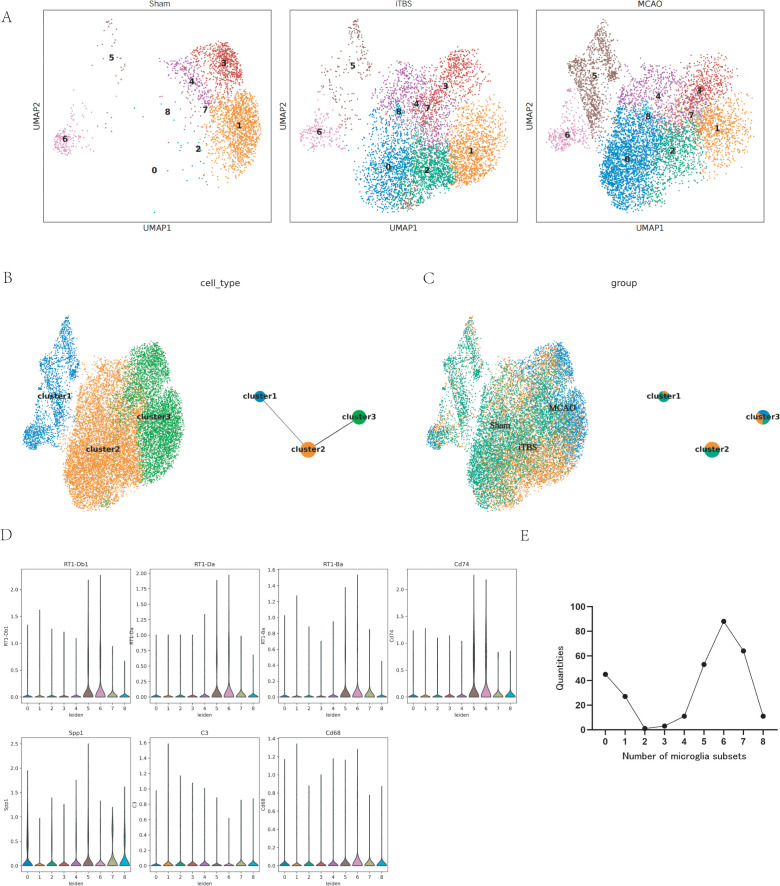
iTBS is associated with changes in the distribution and inflammatory transcriptional features of microglial subpopulations after ischemic stroke. **(A)** UMAP projections of microglial cells from the Sham, iTBS, and MCAO groups, shown separately to illustrate group-specific distributions of microglial subclusters. **(B)** UMAP visualization of reclustered microglia, revealing three major microglial clusters (cluster 1–3) and their transcriptional heterogeneity. The schematic on the right illustrates the inferred relationships among clusters. **(C)** UMAP embedding overlaid with experimental group information, showing differential distribution patterns of microglial clusters across Sham, MCAO, and iTBS conditions. **(D)** Violin plots depicting single-cell expression distributions of inflammation- and immune-associated marker genes (RT1-Db1, RT1-Da, RT1-Ba, Cd74, Spp1, C3, and Cd68) across microglial subclusters. **(E)** Quantification of cell numbers across microglial subclusters, illustrating the relative abundance of each subset within the dataset.Each dot represents a single microglial cell. Violin plots and cell number distributions are shown at the single-cell level without statistical comparison. Differential expression and cell number analyses were performed on aggregated single-cell data as described in the Methods. Differential expression and cell number analyses were performed on aggregated single-cell data as described in the Methods. UMAP distributions, violin plots, and cell-number displays are presented for descriptive visualization and were not used as formal animal-level statistical comparisons.

To investigate how iTBS modulates microglial heterogeneity following cerebral ischemia–reperfusion, we compared the distribution and transcriptional features of microglial subclusters across Sham, MCAO, and iTBS groups using UMAP visualization. As shown in Fig 4A, microglia from the three experimental groups exhibited distinct spatial distributions, indicating that both ischemic injury and iTBS intervention substantially reshaped the composition and organization of microglial subpopulations at the single-cell level.

Under sham conditions, microglia were sparsely distributed and occupied relatively restricted regions of the UMAP space. In contrast, the MCAO group displayed a pronounced expansion and redistribution of multiple microglial subsets, reflecting marked transcriptional diversification and the emergence of distinct microglial states following ischemic injury. Notably, iTBS treatment was associated with a partial shift away from the ischemia-associated redistribution pattern, resulting in a microglial landscape that was distinct from the MCAO group and intermediate between the Sham and MCAO conditions (Fig 4A), suggesting that iTBS modulates microglial state composition rather than inducing a uniform response.

To further characterize microglial heterogeneity, reclustering analysis identified three major microglial clusters, designated cluster 1, cluster 2, and cluster 3 (Fig 4B). The relative spatial separation among these clusters suggested substantial transcriptional divergence within the microglial population. When cluster identity was overlaid with experimental grouping, microglia from the MCAO and iTBS groups exhibited partial overlap but also formed group-enriched regions, indicating that ischemia and iTBS selectively influenced the distribution and relative abundance of specific microglial states (Fig 4C).

Given the central involvement of immune signaling and antigen presentation pathways in post-ischemic neuroinflammation, we next examined the expression of inflammation- and immunity-related genes across microglial subsets. Violin plot analysis revealed that genes associated with antigen presentation and immune activation, including *RT1-Db1*, *RT1-Da*, *RT1-Ba*, and *Cd74*, displayed heterogeneous expression patterns across microglial subsets (Fig 4D). In addition, complement- and activation-associated genes such as *C3*, *Cd68*, and *Spp1* also exhibited subset-specific expression, further highlighting the functional diversity of microglial transcriptional programs.

Finally, we quantified the number of microglia in each subset to assess their relative abundance. As shown in Fig 4E, microglial subsets were unevenly represented, with certain subsets-particularly subset 6-accounting for a disproportionately large fraction of the microglial population. This quantitative imbalance suggests that specific microglial states may become relatively enriched after ischemic injury and could contribute disproportionately to the post-stroke inflammatory microenvironment.

Collectively, these findings suggest that iTBS is associated with changes in the distribution and transcriptional features of multiple microglial states rather than a single binary activation phenotype. Instead, iTBS reshapes the distribution, abundance, and functional transcriptional programs of distinct microglial subsets after cerebral ischemia-reperfusion injury, supporting a heterogeneity-based model of microglial regulation rather than a binary polarization framework.

### Probing and verification of special genes

**Fig 5:**
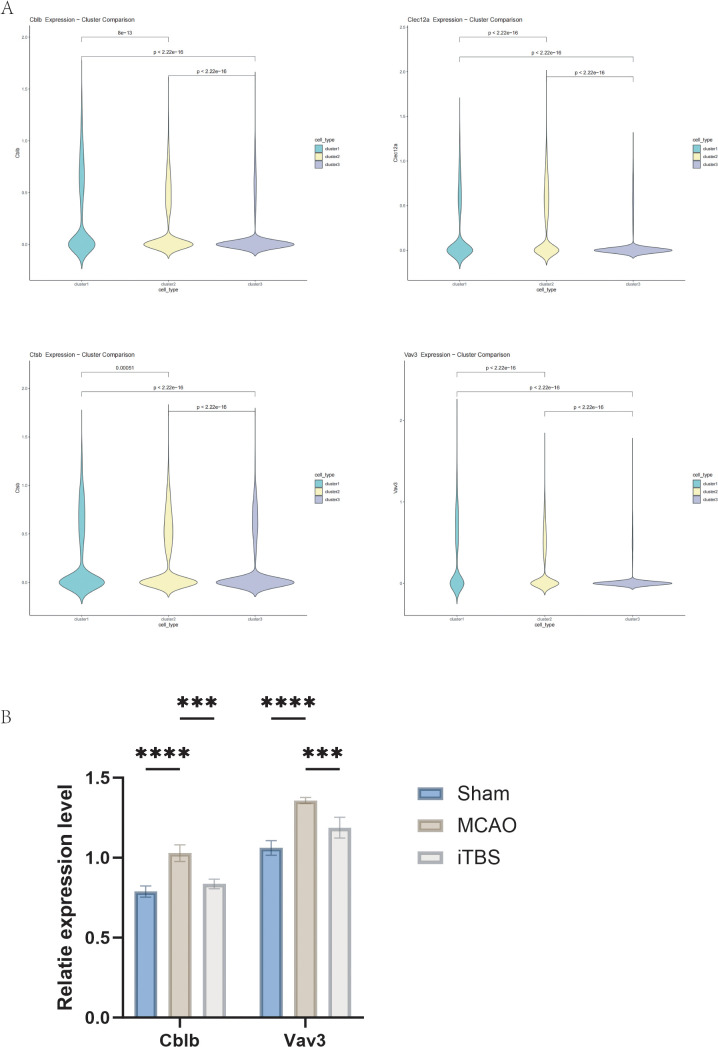
Effect and validation of inflammation-related genes in infarcted brain tissue following iTBS intervention. **(A)** Differential expression of inflammation-related genes (*Cblb*, *Vav3*, *Clec12a*, and *Ctsb*) identified from microglial transcriptomic analysis in infarcted brain tissues from rats in the Sham, MCAO, and iTBS groups. **(B)** RT–qPCR validation of *Cblb* expression in homogenized infarcted brain tissues from rats subjected to different treatments. **(C)** RT–qPCR validation of *Vav3* expression in homogenized infarcted brain tissues from rats subjected to different treatments.Data are presented as mean ± SEM. Each data point represents one independent animal (n = 3 animals per group). Statistical analyses were performed at the animal level using the methods described in the Methods section. ***P < 0.001,****P < 0.0001.

To investigate whether iTBS has an inhibitory effect on inflammation in the brain tissue microenvironment, we analyzed the transcriptomic data of microglia and investigated DEGs after different interventions. The observed downregulation of *Cblb*, *Vav3*, *Clec12a*, and *Ctsb* following iTBS treatment suggests a potential association between iTBS and altered inflammatory gene expression in microglia. However, these transcriptional changes alone do not establish causal regulatory relationships. Instead, they identify candidate genes whose functional roles in post-ischemic neuroinflammation warrant further investigation using targeted genetic or pharmacological approaches. (Fig 5A). Reduced expression of these genes was observed in the iTBS group relative to MCAO. Among them, *Cblb* (E3 ubiquitin ligase) has the function of negatively regulating T/B cell receptor signaling and preventing immune over-activation; it is involved in the regulation of the (Toll-like receptor 4)TLR4/NF-κB pathway. The reduced expression of Cblb observed after iTBS may be consistent with altered inflammatory signaling, although the present data do not establish whether Cblb directly mediates these downstream effects. *Vav3* (guanine nucleotide exchange factor) regulates Rho GTPase activity and affects cell migration and phagocytosis; it is involved in B cell receptor and CXCR4 signaling. Reduced *Vav3* expression may be associated with altered migratory or signaling properties of microglia, but this interpretation remains speculative. *Clec12a* (inhibitory C-type lectin receptor) recognizes danger signals, such as uric acid crystallization, and transmits inhibitory signals through SHP-1/2; however, it may promote inflammation through the Syk pathway in some cases. However, the classical theory is that *Clec12a* inhibits inflammation and its downregulation may enhance inflammation. This needs to be judged in the context of other gene expressions and pathology and may be explained by the fact that in the ischemic microenvironment, *Clec12a* may activate NLRP3 inflammatory vesicles through the *Syk* pathway, which is blocked by the downregulation of iTBS. *Ctsb* (Cathepsin B) lysosomal protease, which activates NLRP3 inflammatory vesicles, promotes the maturation of Interleukin-1beta (IL-1β). The observed *Ctsb* decrease raises the possibility of altered inflammasome-related signaling, but direct functional effects were not tested in the present study. Among them, gene *Cblb* and gene *Vav3* were ranked 32nd and 95th among the 496 differentially expressed genes in MG6, respectively.

Next, in order to further verify the specificity of these genes, we extracted RNA from different treated rat infarcted area brain tissues after polishing them into homogenate, and we performed rt-qPCR to verify the RNA after converting it into cDNA for different groups of treatments.

## Discussion

In this study, we employed a comprehensive approach integrating transcriptome analysis and bioinformatics tools to elucidate the major brain cell types and their cell-type-specific genes in ischemic stroke. Our study provides a detailed analysis of ischemic stroke-associated genes and pathways. Recent authoritative reviews have highlighted that rTMS and iTBS can function as systemic immunomodulatory interventions, capable of reshaping neuroinflammatory cascades beyond purely neurophysiological effects. Studies emphasize that neuromodulation-induced immune regulation—especially via microglia—may represent a key mechanism underlying functional recovery after stroke and other neurological disorders [8–10].

In this context, our single-cell data provide cell-resolved descriptive evidence consistent with the possibility that iTBS is associated with altered microglial transcriptional programs related to inflammation resolution, phagocytosis, and tissue repair.

Specifically, we characterize unique gene expression profiles in microglia subpopulations and reveal significant heterogeneity among other brain cell types post-ischemic injury. Recent research has highlighted the ability of transcranial magnetic stimulation (TMS) to influence the nervous system, specifically through its impact on microglia, which can shift to an M2 phenotype [23]. Among the various TMS protocols, intermittent Theta Burst Stimulation (iTBS) is a distinct approach, although the underlying mechanisms of this technique are not fully understood. Microglia, the brain’s resident immune cells, act as the central nervous system’s primary defense. In their resting state (M0 phenotype), they monitor the environment through immunosurveillance. However, when faced with pathology, microglia rapidly activate and undergo transcriptional changes. M1-polarized microglia, which are classically activated, release pro-inflammatory substances and cytotoxic agents to eliminate pathogens, while the M2-polarized microglia, which are alternatively activated, play a critical role in repairing tissue and promoting neuroprotection through regeneration. While the M1/M2 framework has historically provided a useful descriptive model, it is now widely regarded as an oversimplification. Single-cell studies over the past decade have revealed substantial microglial heterogeneity, with overlapping, context-dependent, and transitional states that cannot be fully captured by binary classifications. Accordingly, references to ‘M1-like’ or ‘M2-like’ states in this manuscript are intended as descriptive shorthand for dominant transcriptional programs rather than fixed microglial identities.

Despite the inclusion of multiple brain cell types in the single-cell dataset, the present study does not provide equally detailed mechanistic analyses for astrocytes, oligodendrocyte lineage cells, or vascular-associated cells. Accordingly, interpretations regarding these populations should be considered hypothesis-generating rather than definitive. Previous single-cell studies have revealed substantial heterogeneity and functional specialization within astrocytes and oligodendrocyte precursor cells after ischemic injury [24], underscoring the need for future studies employing cell-type–specific transcriptomic analyses, spatial transcriptomics, or cell–cell interaction modeling to fully elucidate their contributions to iTBS-mediated recovery.

Our data are consistent with the possibility that iTBS is associated with a redistribution of microglial transcriptional programs toward reparative and inflammation-resolving states, which may contribute to an attenuated neuroinflammatory milieu. Normally, neuroinflammation functions as a biological defense mechanism responding to infection or injury; however, when this reaction becomes excessive, it leads to neuronal damage. Microglial programs commonly described as repair-associated or inflammation-resolving may help counterbalance excessive inflammatory injury. This phenotypic transformation also activates several endogenous repair pathways. As an intrinsic recovery process of the organism, endogenous repair in the nervous system relies on M2 microglia to create a supportive microenvironment for neuronal regeneration. By releasing neurotrophic factors, these cells foster the proliferation and differentiation of neural stem cells and facilitate the restoration of injured neural tissues [25].

In addition, the present study suggests candidate transcriptional programs and cellular patterns potentially associated with microglial state changes after ischemic injury and iTBS intervention: monocyte infiltration and excessive engagement of pro-inflammatory microglial programs in the acute phase [26] (MCAO group) may exacerbate neuroinflammation [27] through the release of TNF-α/IL-1β, leading to massive neuronal death and disruption of the blood-brain barrier, along with abnormal astrocyte. In contrast, during the repair phase (iTBS group), iTBS treatment was associated with a redistribution of microglial transcriptional states toward programs linked to immune regulation and tissue repair, accompanied by reduced expression of genes related to monocyte chemotaxis, including *Ccl2* and integrin-associated pathways, cooperated with a high proportion of astrocytes to secrete repair factors such as IL-10/GDNF, activated PI3K/Akt anti-apoptotic pathway and Shh/Notch regenerative signaling, and promoted vascularization (VEGF-mediated) and oligodendrocyte precursor cell proliferation, potentially contributing to a tissue environment permissive for neuronal survival and remodeling. This process involves multi-level mechanisms such as inflammation-repair balance regulation, glial-immune cell interaction, and microenvironmental homeostasis reconstruction.

The methodology used in this study has significant advantages that improve the robustness and reliability of the results. By utilizing advanced single-cell RNA sequencing technology, we could comprehensively characterize the major brain cell types and their associated gene expression profiles, which are critical for understanding the cellular dynamics of ischemic stroke. By comprehensively evaluating differentially expressed genes and the pathways they are involved in, we can gain insight into the molecular basis of ischemic injury and thus identify potential therapeutic targets. Furthermore, the exploration of unique gene expression profiles within microglia subpopulations highlights the intricate heterogeneity of immune responses in the brain, and this multifaceted study not only elucidates the complex interplay of cellular responses to ischemic injury but also lays the groundwork for future studies of targeted interventions. A noteworthy limitation is the reliance on a specific ischemic injury model that may not fully encapsulate the complexity of human stroke pathology. Brain cell types and their responses to ischemic conditions may differ significantly across species, and thus the translational applicability of our findings may be limited. In addition, although we identified unique gene expression signatures of microglia subpopulations, the functional significance of these signatures remains to be elucidated, necessitating further studies. The relatively small sample size used in assessing differentially expressed genes and associated pathways may limit the robustness and generalizability of the findings. Future studies should aim to incorporate larger cohorts and different models to validate these findings and explore the intricate cell-cell communication networks that emerge after ischemic events. The diversity of cell-cell communication after brain injury and the dynamic interactions between cell types may influence recovery and repair processes. This part of the study needs to be refined. These will not only improve our understanding of cellular conditions after ischemic events but will also pave the way for targeted therapeutic strategies aimed at modulating these interactions to improve the prognosis of stroke patients.

In the present study, *Cblb* and *Vav3* emerged as candidate MG6-associated genes in our dataset and may represent potentially interesting targets for future investigation in ischemic neuroinflammation. Notably, the known functions of these two genes in ubiquitylation modification *(Cblb*) and chemokine receptor signaling (*Vav3)* suggest that the MG6 subpopulation may participate in the neuroinflammatory cascade following cerebral infarction through a distinct immunomodulatory pathway involved in the neuroinflammatory cascade response after cerebral infarction. In contrast to previous studies, the present findings extend the theoretical framework of Anca et al. [28] regarding the role of *Vav3* in macrophage migration, while providing new cell type-specific evidence for the regulatory mechanism of Casitas B lineage lymphoma protein (*Cblb*) in CNS inflammation [29]. However, these results should be framed as hypothesis-generating. The differential expression of genes like *Cblb* and *Vav3* indicates potential roles in immune regulation and recovery, but further studies are needed to validate their functional significance in vivo.

Through RT-qPCR validation based on tissue homogenates from infarcted areas, qPCR analysis in an independent set of biological samples showed expression trends for *Cblb* and *Vav3* that were directionally consistent with the scRNA-seq observations, providing limited orthogonal support for these candidate genes (p < 0.05, two-tailed t-test). This mutual validation of expression patterns across technological platforms not only confirms the detection reliability of the target genes but more importantly reveals the detectability characteristics of microglia subpopulation-specific gene expression at the tissue level. However, it should be pointed out that the tissue homogenization method may result in reduced cell type resolution, which suggests that subsequent studies need to be combined with laser capture microdissection or flow sorting techniques for cell subpopulation-specific validation.

Based on prior literature and the transcriptional patterns observed here, one possible interpretation is that *Cblb* and *Vav3* may be linked to signaling processes relevant to microglial activation and leukocyte recruitment. However, the present study does not establish causal roles for these genes in vivo. This forms a spatial-functional coupling with our observation that the high-density distribution of MG6 subpopulations is characterized in the peri-infarct zone. However, the present study has not yet elucidated the temporal expression pattern of these two genes in microglia phenotypic transition, and future conditional knockout animal models will be required to verify their causal roles.

Several limitations should be acknowledged. First, the number of biological replicates for single-cell sequencing was modest, which constrains statistical power for pathway-level inference. Second, pathway enrichment analyses were based on transcriptomic associations and do not capture post-transcriptional regulation or functional pathway activity. Future studies incorporating larger cohorts, longitudinal sampling, and functional validation experiments will be essential to test the hypotheses generated by the present dataset. Third，notably, the absence of in-depth analyses for non-microglial populations should not be interpreted as evidence of biological irrelevance; rather, the observed compositional changes in astrocytes, oligodendrocyte lineage cells, and vascular-associated cells highlight potentially important multicellular responses that could not be resolved at sufficient resolution within the scope of the present dataset.

In addition, the sham stimulation paradigm used in this study may not fully control for non-specific sensory and environmental effects associated with iTBS. Although the coil was positioned 15 cm above the animal’s head to avoid effective magnetic stimulation, this approach does not completely eliminate potential confounding factors such as acoustic noise, mechanical vibration, or handling-related stress. These non-specific factors could partially contribute to the observed behavioral or molecular changes. Therefore, future studies employing more refined sham controls—such as angled coils, shielded coils, or active sham devices that better mimic sensory inputs without inducing cortical stimulation—will be important to further validate the specificity of iTBS effects.

## Conclusion

In summary, our findings highlight the complexity of cellular responses to ischemic stroke, highlighting the distinct gene expression profiles of the major brain cell types, particularly the unique characteristics of microglia subpopulations. The comprehensive assessment of differentially expressed genes and associated pathways highlights candidate transcriptional changes and cellular patterns associated with ischemic injury, suggesting that cellular heterogeneity may contribute to stroke pathophysiology.

## Supporting information

S1 Fig Characterization of the Y064 coil and its electromagnetic field properties. (A) Schematic illustration of the placement position of the Y064 coil relative to the experimental subject. (B) Simulated magnetic field distribution generated by the Y064 coil. (C) Simulated electric field distribution generated by the Y064 coil. (D) Structural design and geometric configuration of the Y064 coil. (E) Electric field intensity as a function of distance (0–6 cm) measured perpendicularly from the coil surface. The electric field strength can be estimated according to the actual or predicted distance between the coil and the cerebral cortex of the subject. The coil geometry and stimulation parameters used in the COMSOL simulation were identical to those described in the experimental setup. The spatial distribution of the induced electric field and its decay with distance are shown in S1 Fig, allowing estimation of field strength at the cortical level.
(PDF)

S2 Fig Quality control metrics of single-cell RNA sequencing data before and after filtering. Each point represents an individual cell. (A) Quality control metrics before filtering. (B) Quality control metrics after filtering. nGene plot: Distribution of the number of detected genes per cell across samples. nUMI plot: Distribution of the number of unique molecular identifiers (UMIs) detected per cell across samples. percent.mt plot: Distribution of the percentage of mitochondrial gene expression per cell across samples.
(PDF)

S3 Fig Correlation analysis of single-cell RNA sequencing quality control metrics. Each point represents an individual cell. The Pearson correlation coefficient is displayed at the top of each panel. The left panel shows the relationship between the number of UMIs detected per cell and the percentage of mitochondrial gene expression. The right panel shows the relationship between the number of UMIs detected per cell and the number of expressed genes per cell.
(PDF)

S4 Fig Characterization of microglial subfamilies based on gene expression profiles. (A) Violin plots displaying the top five signature genes for each identified microglial subfamily. (B) Bubble plot illustrating the top three signature genes for each microglial subfamily. Bubble size represents the proportion of cells expressing the gene, and color intensity indicates the relative expression level.
(PDF)

S1 File This zip file contains supplementary datasets, including raw data from open field behavioral assays, qRT-PCR experiments, and single-cell RNA sequencing. These materials provide additional support for the results presented in this study.
(ZIP)
