# Supplementary figures and images for: Single-cell RNA sequencing identifies microglial state changes associated with iTBS after ischemia–reperfusion injury
Source: PLoS One. 2026 Apr 20;21(4):e0346888. doi: 10.1371/journal.pone.0346888 (PMC13095131; doi:10.1371/journal.pone.0346888)

A

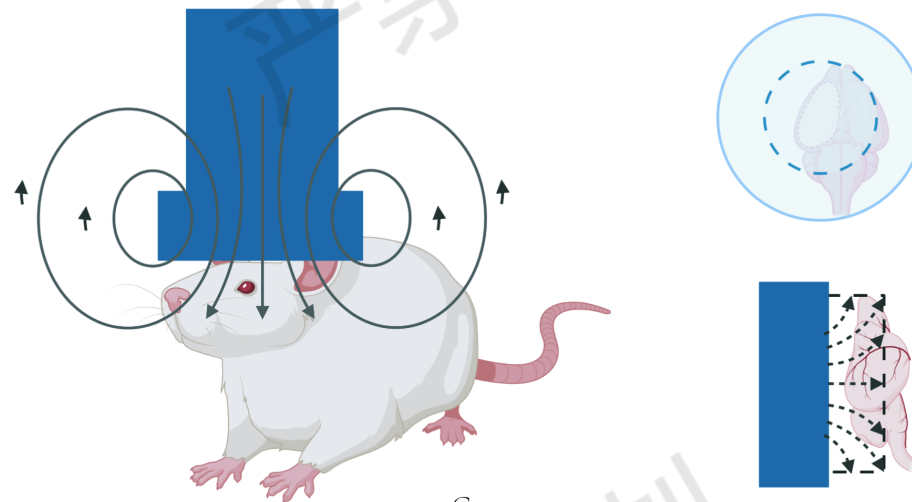

B

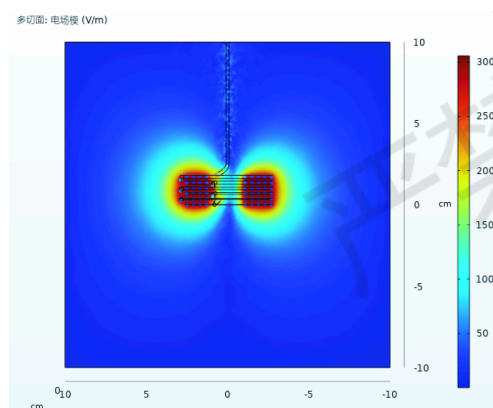

C

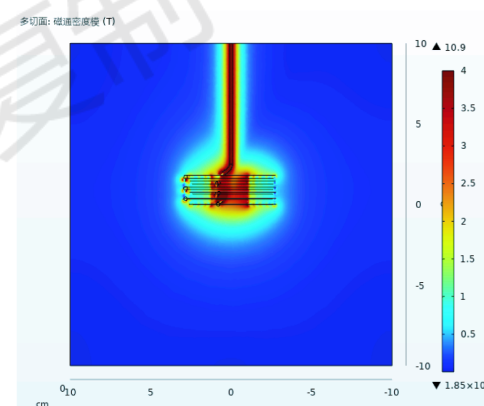

D

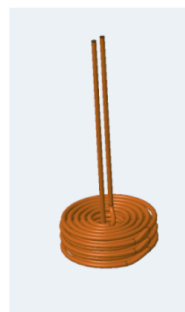

E

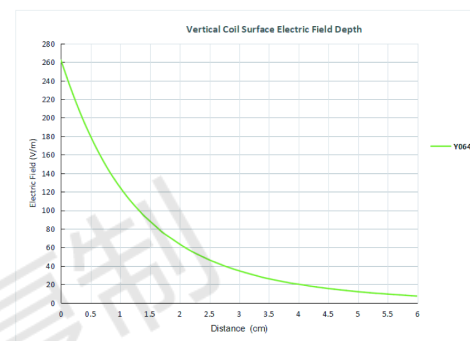

Supplement: S1 Fig — (A) Schematic illustration of the placement position of the Y064 coil relative to the experimental subject. (B) Simulated magnetic field distribution generated by the Y064 coil. (C) Simulated electric field distribution generated by the Y064 coil. (D) Structural design and geometric configuration of the Y064 coil. (E) Electric field intensity as a function of distance (0–6 cm) measured perpendicularly from the coil surface. The electric field strength can be estimated according to the actual or predicted distance between the coil and the cerebral cortex of the subject. The coil geometry and stimulation parameters used in the COMSOL simulation were identical to those described in the experimental setup. The spatial distribution of the induced electric field and its decay with distance are shown in S1 Fig, allowing estimation of field strength at the cortical level. (PDF) [file pone.0346888.s001.pdf]

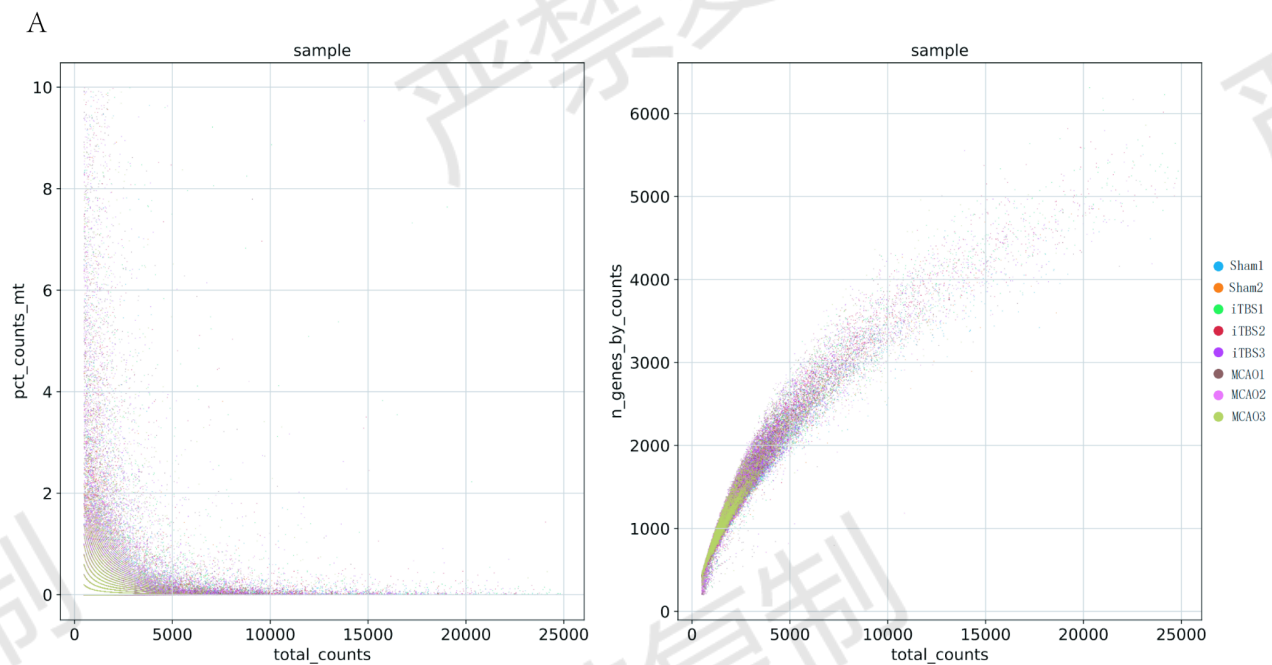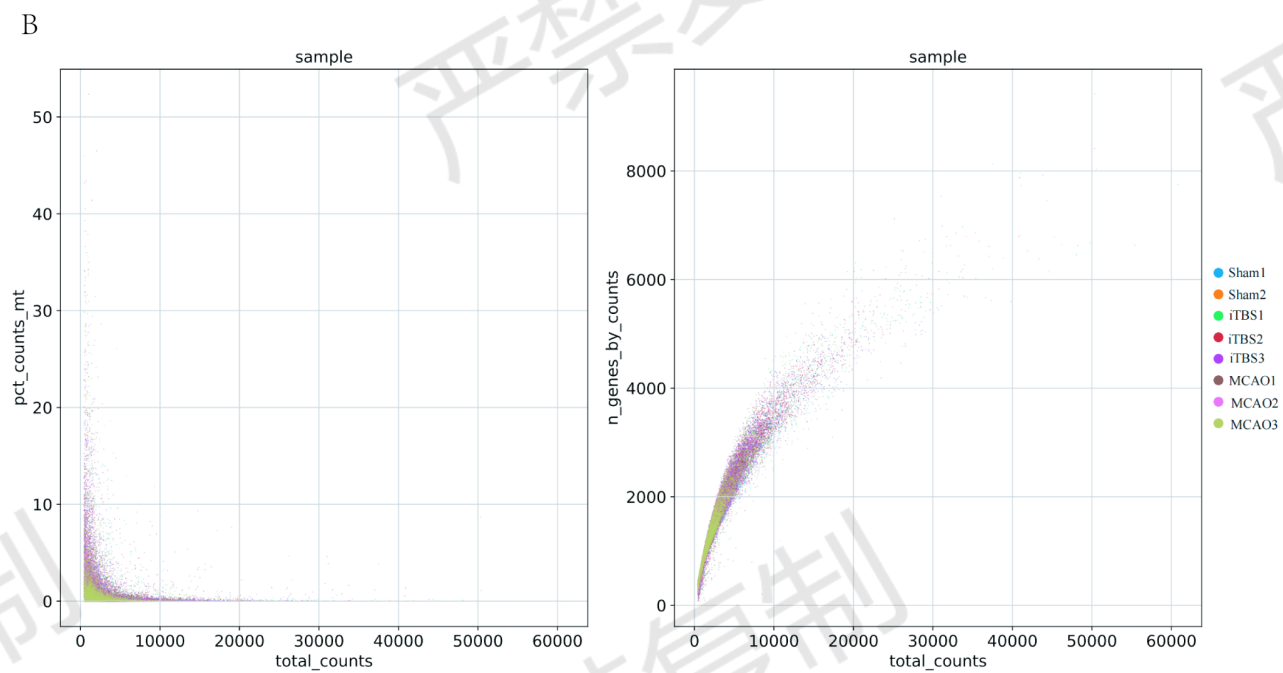

Supplement: S2 Fig — Each point represents an individual cell. (A) Quality control metrics before filtering. (B) Quality control metrics after filtering. nGene plot: Distribution of the number of detected genes per cell across samples. nUMI plot: Distribution of the number of unique molecular identifiers (UMIs) detected per cell across samples. percent.mt plot: Distribution of the percentage of mitochondrial gene expression per cell across samples. (PDF) [file pone.0346888.s002.pdf]

A

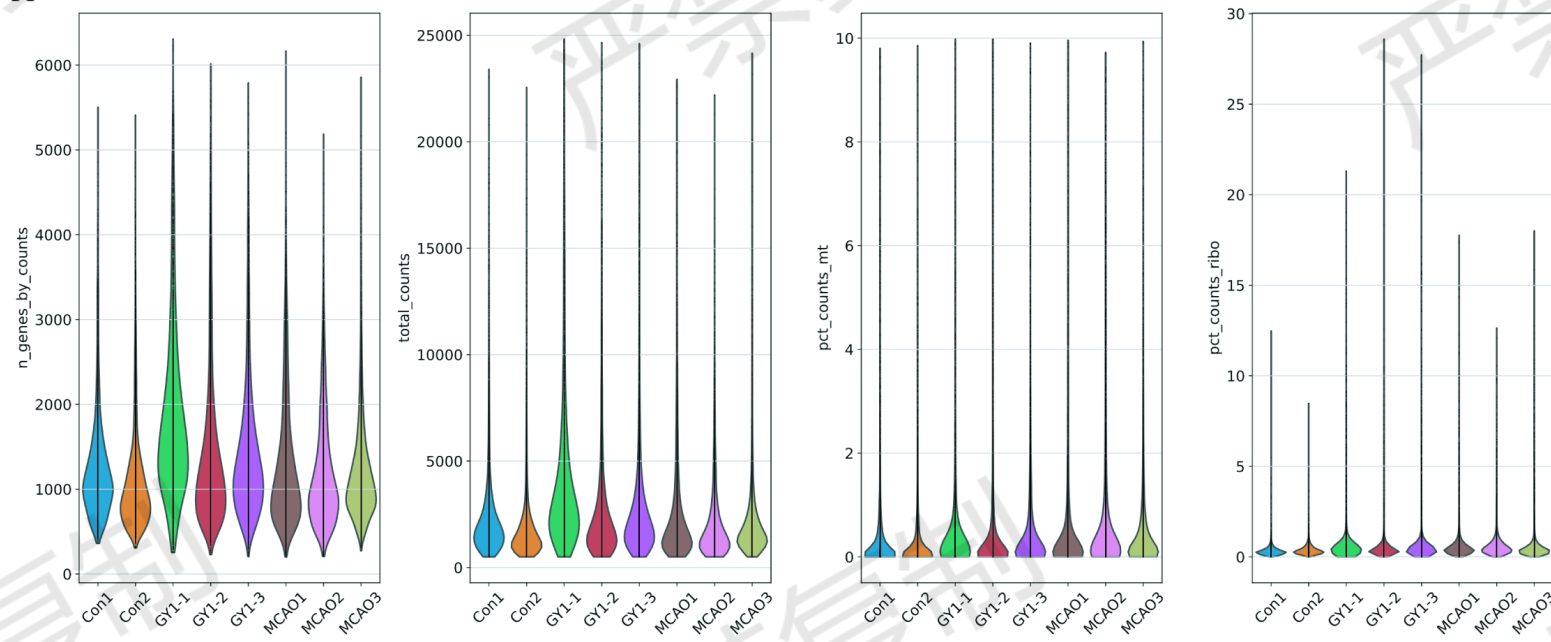

B

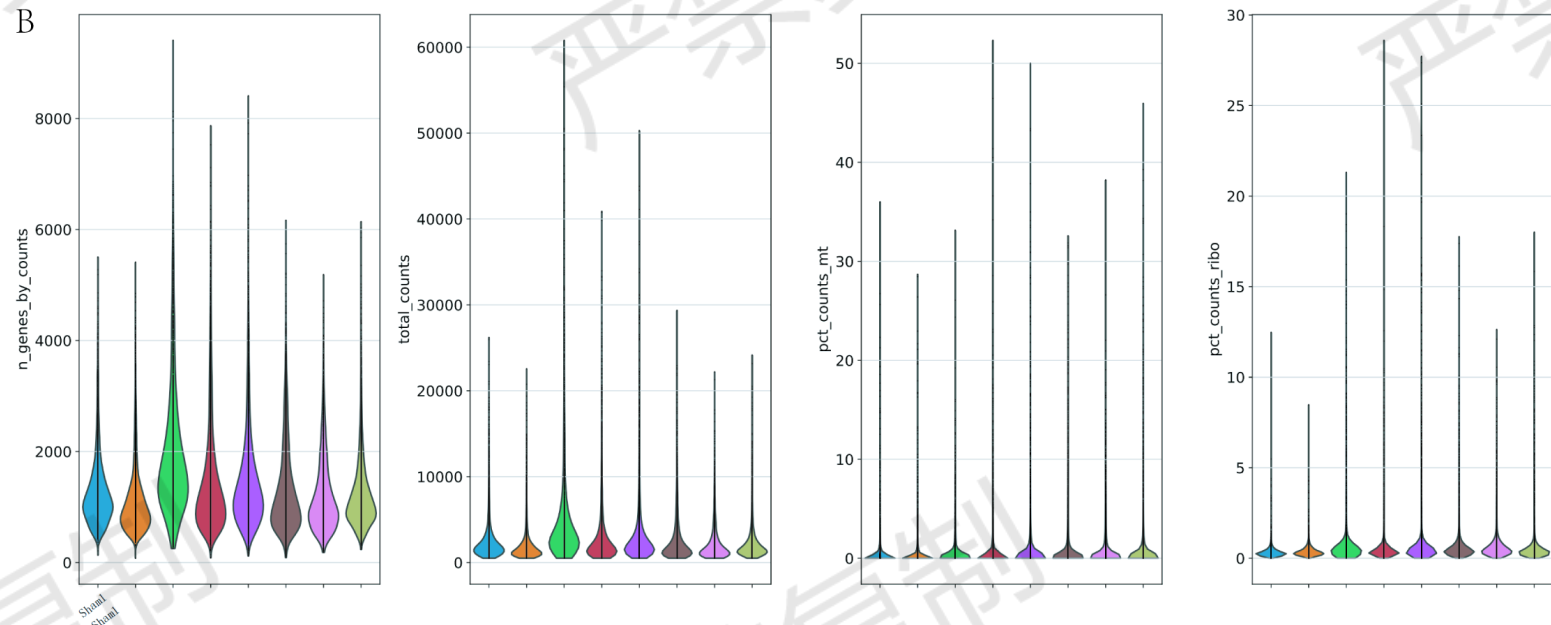

Supplement: S3 Fig — Each point represents an individual cell. The Pearson correlation coefficient is displayed at the top of each panel. The left panel shows the relationship between the number of UMIs detected per cell and the percentage of mitochondrial gene expression. The right panel shows the relationship between the number of UMIs detected per cell and the number of expressed genes per cell. (PDF) [file pone.0346888.s003.pdf]

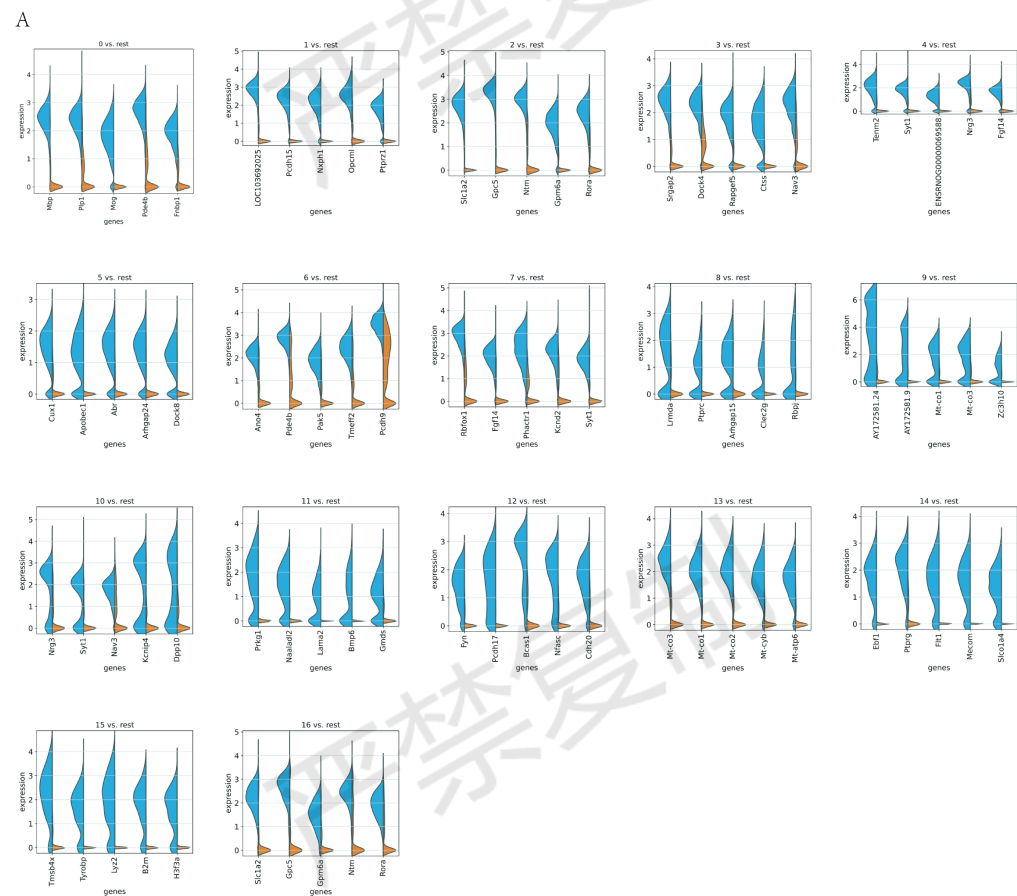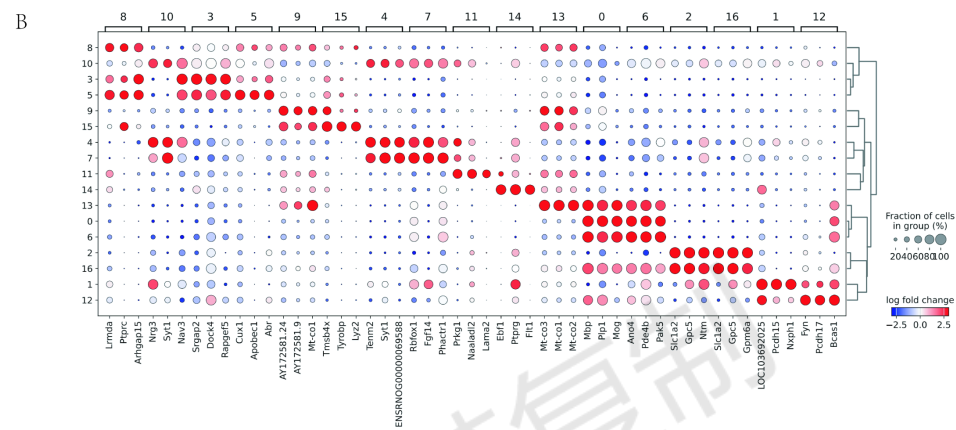

Supplement: S4 Fig — (A) Violin plots displaying the top five signature genes for each identified microglial subfamily. (B) Bubble plot illustrating the top three signature genes for each microglial subfamily. Bubble size represents the proportion of cells expressing the gene, and color intensity indicates the relative expression level. (PDF) [file pone.0346888.s004.pdf]

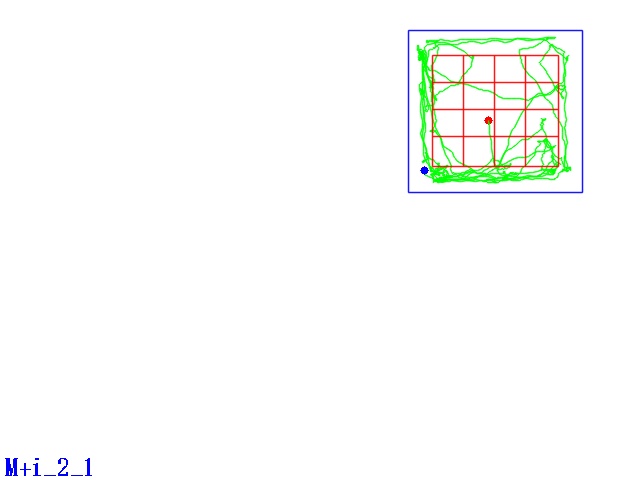

Supplement: S1 File — These materials provide additional support for the results presented in this study. (ZIP) [file pone.0346888.s005.zip › Open Field_Figure/Figure_iTBS/M+i-2-1_20241008203636.jpg]

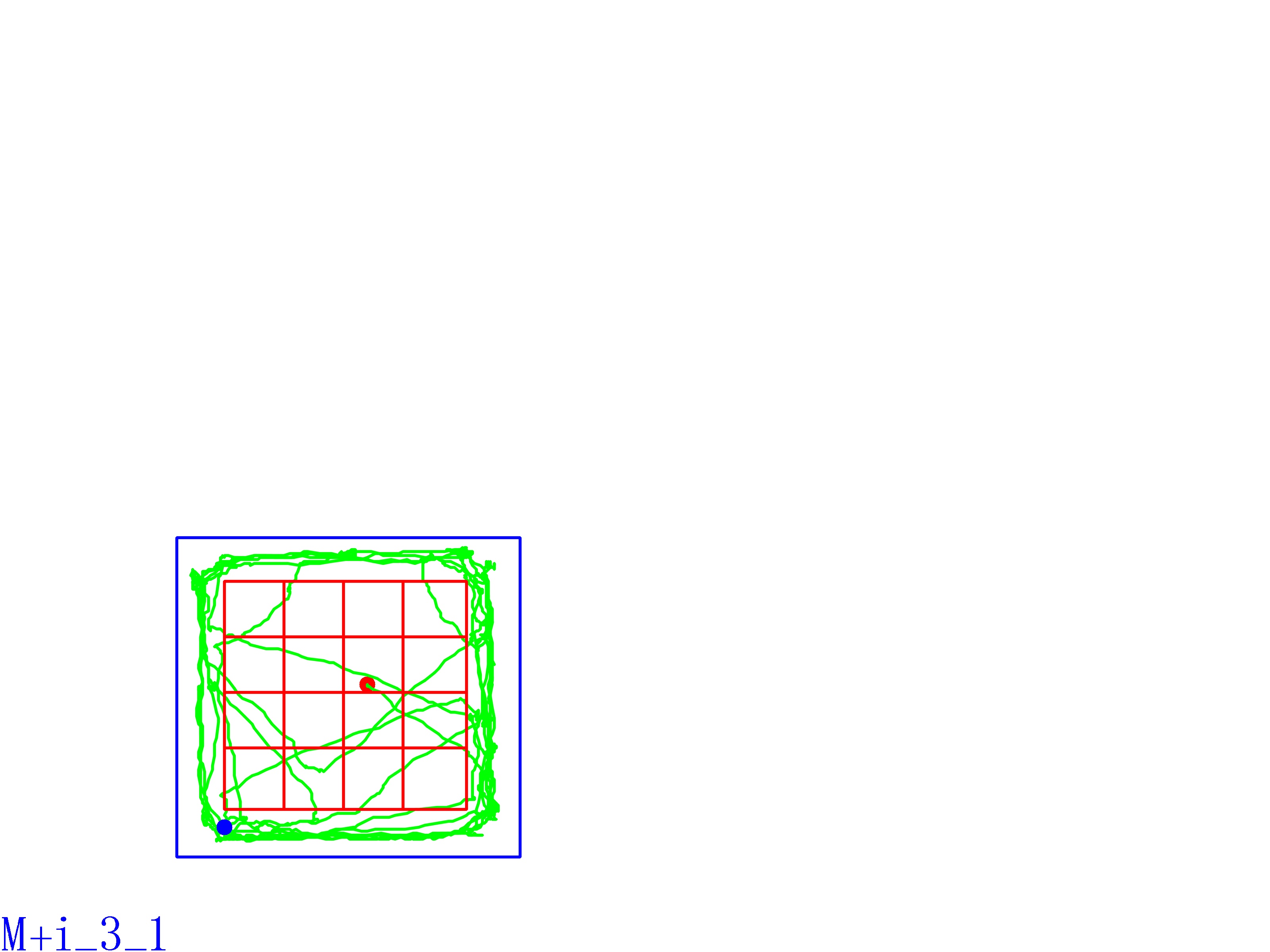

Supplement: S1 File — These materials provide additional support for the results presented in this study. (ZIP) [file pone.0346888.s005.zip › Open Field_Figure/Figure_iTBS/M+i-3-1_20241008203636.jpg]

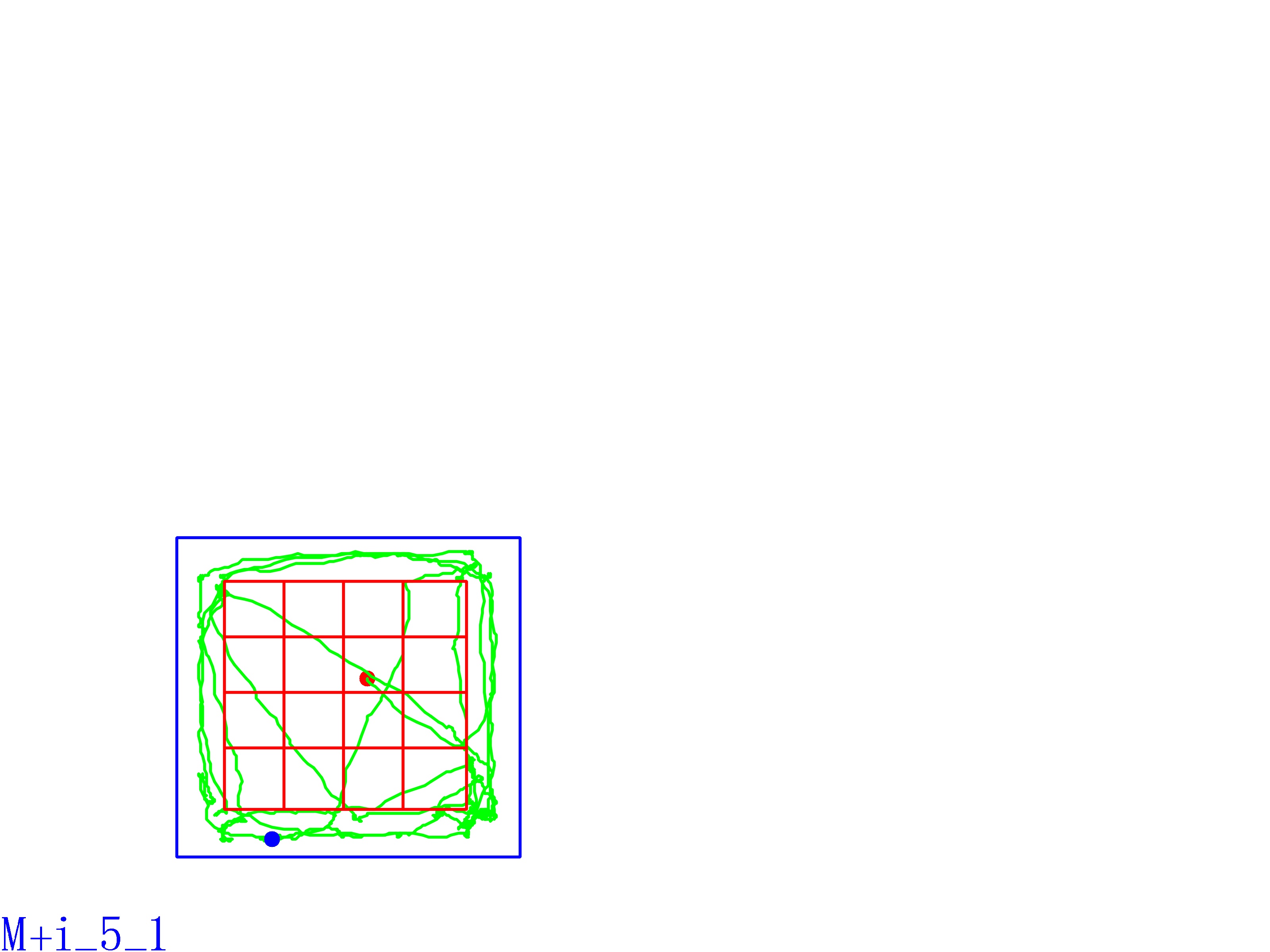

Supplement: S1 File — These materials provide additional support for the results presented in this study. (ZIP) [file pone.0346888.s005.zip › Open Field_Figure/Figure_iTBS/M+i-5-1_20241008203637.jpg]

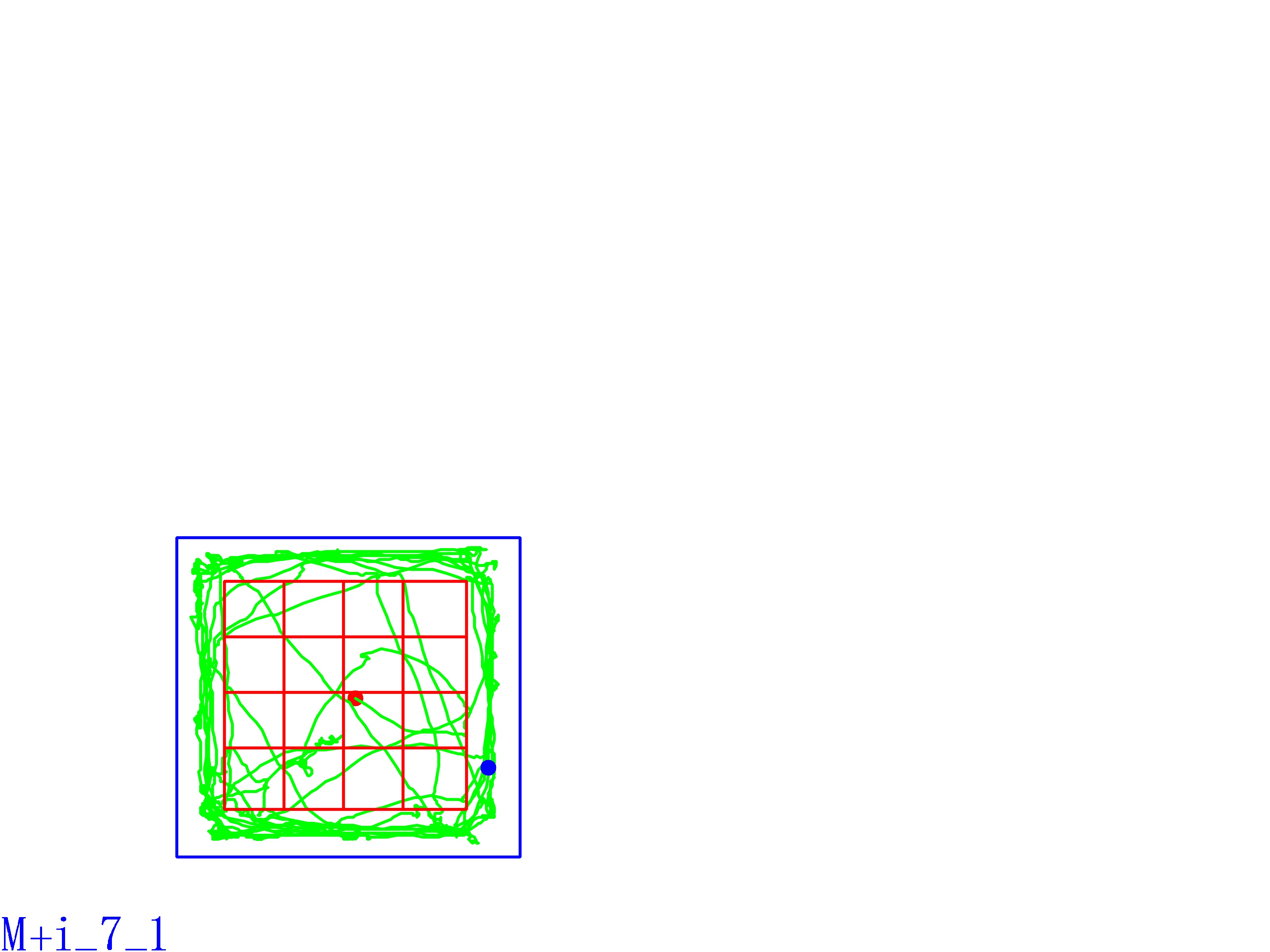

Supplement: S1 File — These materials provide additional support for the results presented in this study. (ZIP) [file pone.0346888.s005.zip › Open Field_Figure/Figure_iTBS/M+i-7-1_20241008203637.jpg]

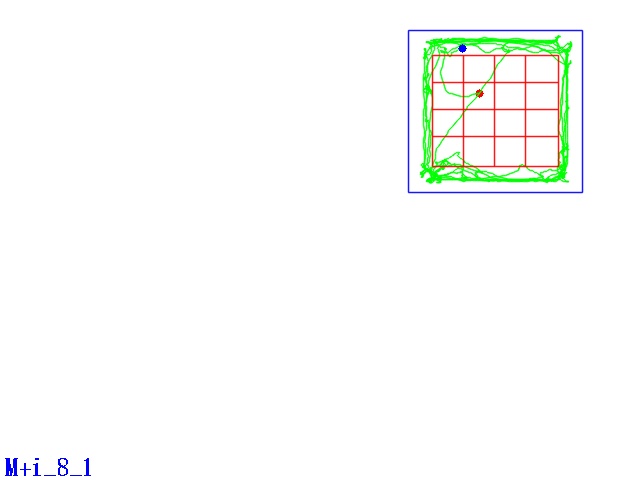

Supplement: S1 File — These materials provide additional support for the results presented in this study. (ZIP) [file pone.0346888.s005.zip › Open Field_Figure/Figure_iTBS/M+i-8-1_20241008203637.jpg]

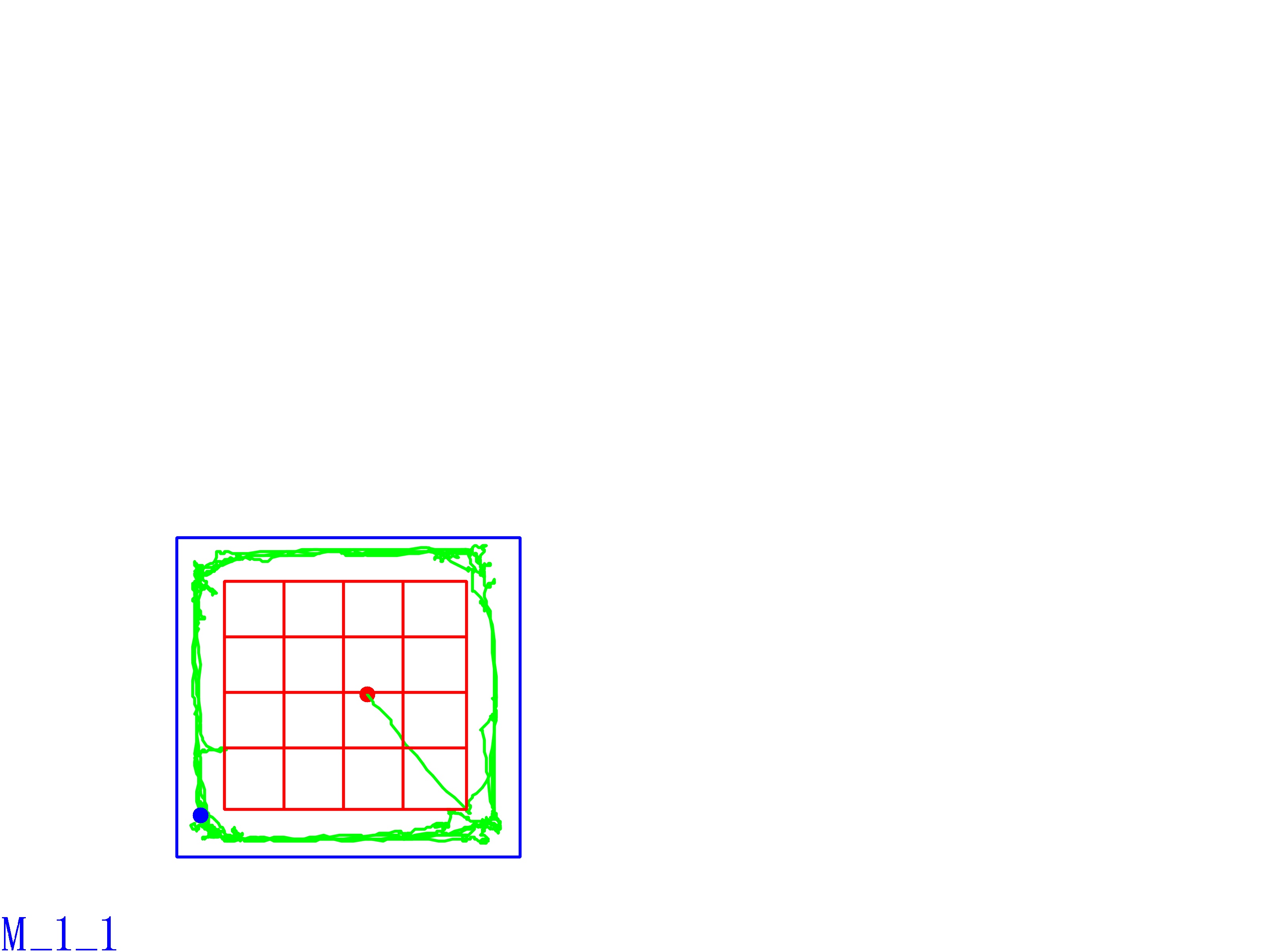

Supplement: S1 File — These materials provide additional support for the results presented in this study. (ZIP) [file pone.0346888.s005.zip › Open Field_Figure/Figure_MCAO/M-1-1_20241008203634.jpg]

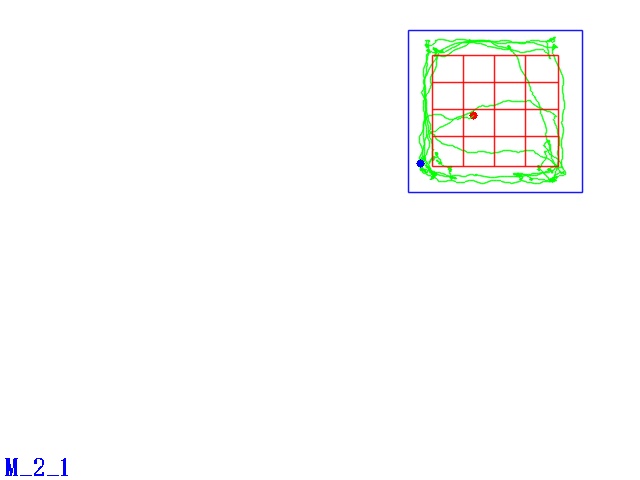

Supplement: S1 File — These materials provide additional support for the results presented in this study. (ZIP) [file pone.0346888.s005.zip › Open Field_Figure/Figure_MCAO/M-2-1_20241008203634.jpg]

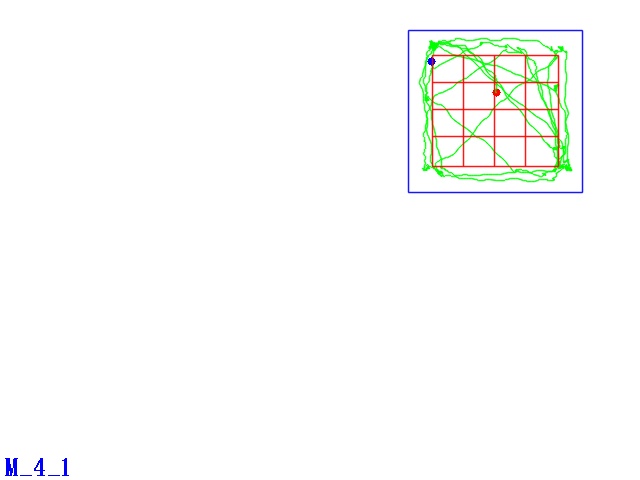

Supplement: S1 File — These materials provide additional support for the results presented in this study. (ZIP) [file pone.0346888.s005.zip › Open Field_Figure/Figure_MCAO/M-4-1_20241008203634.jpg]

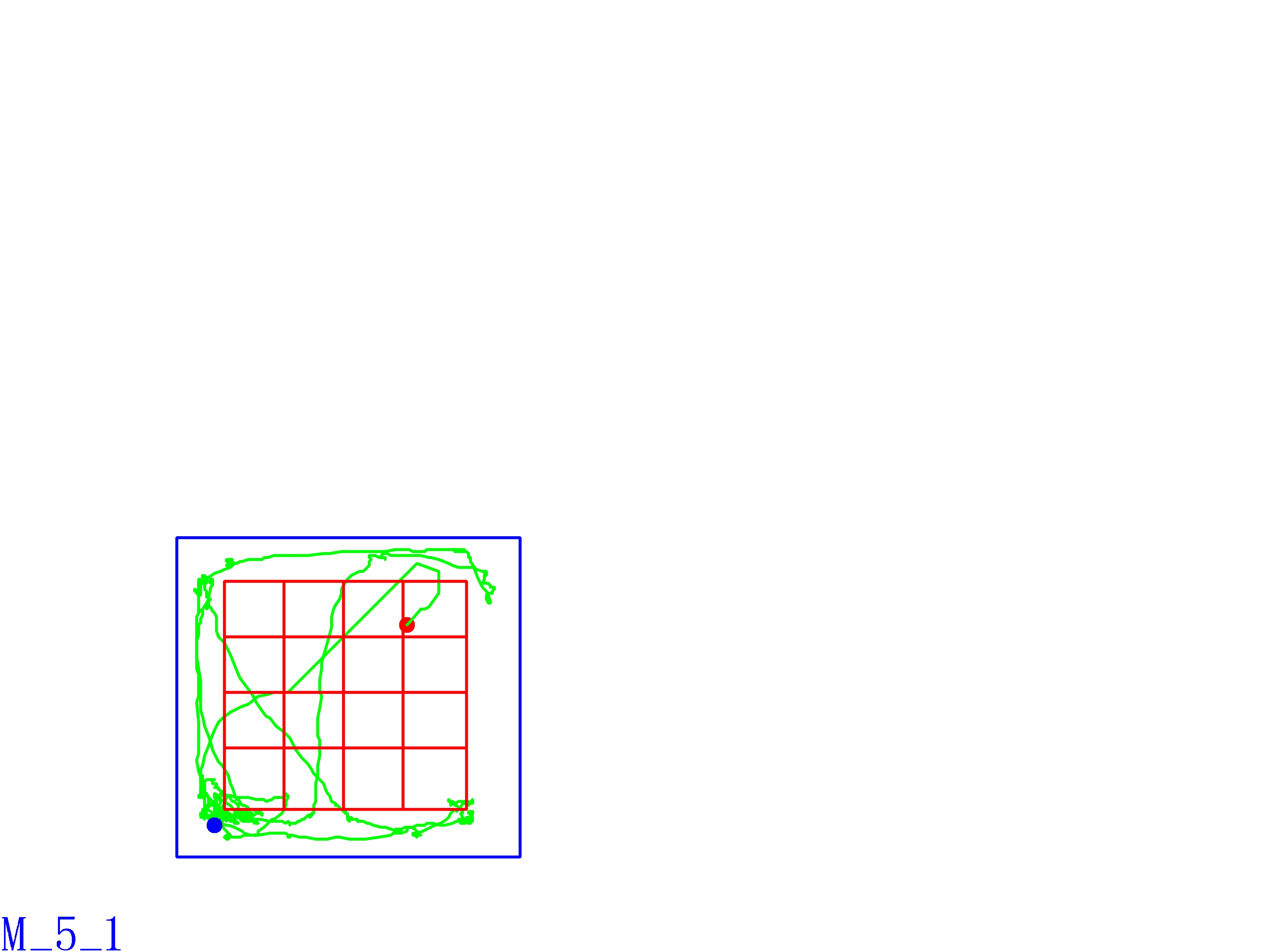

Supplement: S1 File — These materials provide additional support for the results presented in this study. (ZIP) [file pone.0346888.s005.zip › Open Field_Figure/Figure_MCAO/M-5-1_20241008203635.jpg]

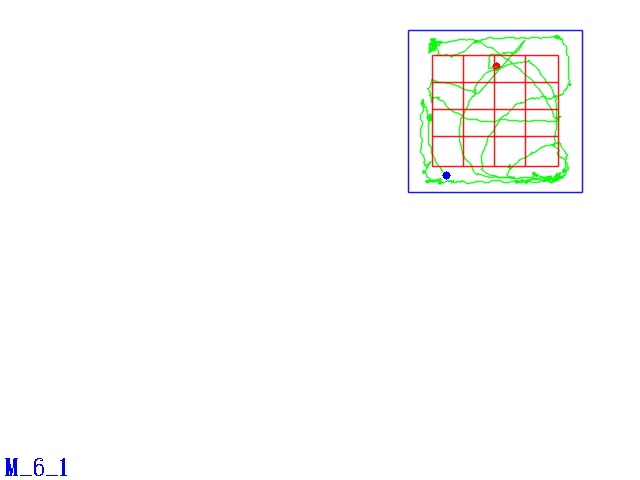

Supplement: S1 File — These materials provide additional support for the results presented in this study. (ZIP) [file pone.0346888.s005.zip › Open Field_Figure/Figure_MCAO/M-6-1_20241008203635.jpg]

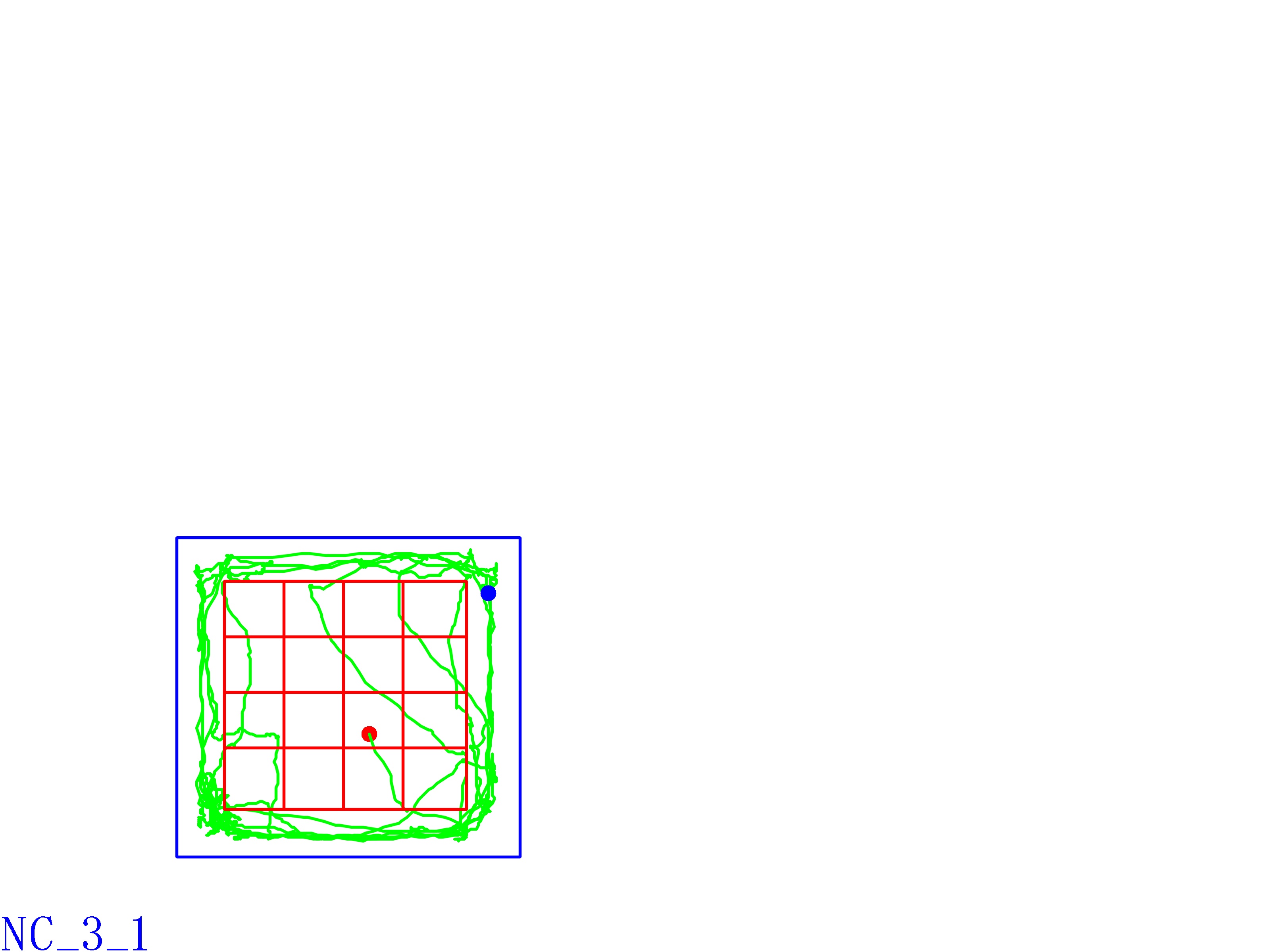

Supplement: S1 File — These materials provide additional support for the results presented in this study. (ZIP) [file pone.0346888.s005.zip › Open Field_Figure/Figure_Sham/S-3-1_20241008203632.jpg]

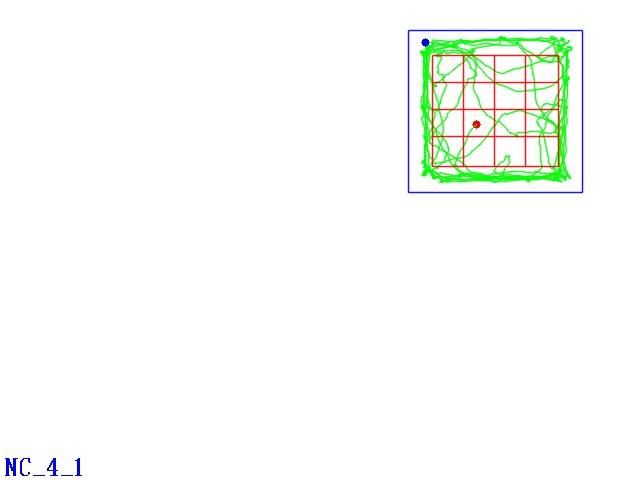

Supplement: S1 File — These materials provide additional support for the results presented in this study. (ZIP) [file pone.0346888.s005.zip › Open Field_Figure/Figure_Sham/S-4-1_20241008203632.jpg]

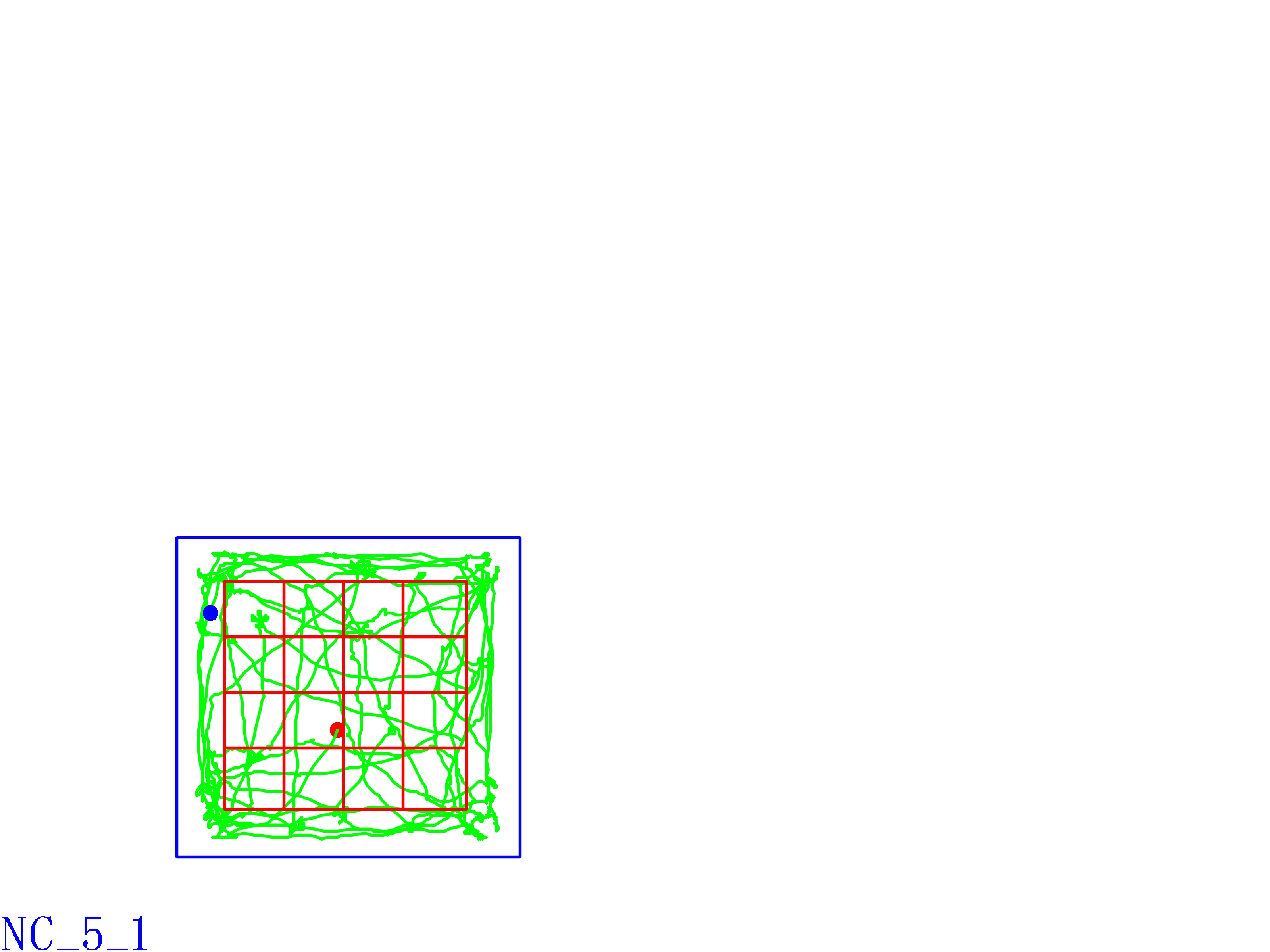

Supplement: S1 File — These materials provide additional support for the results presented in this study. (ZIP) [file pone.0346888.s005.zip › Open Field_Figure/Figure_Sham/S-5-1_20241008203633.jpg]

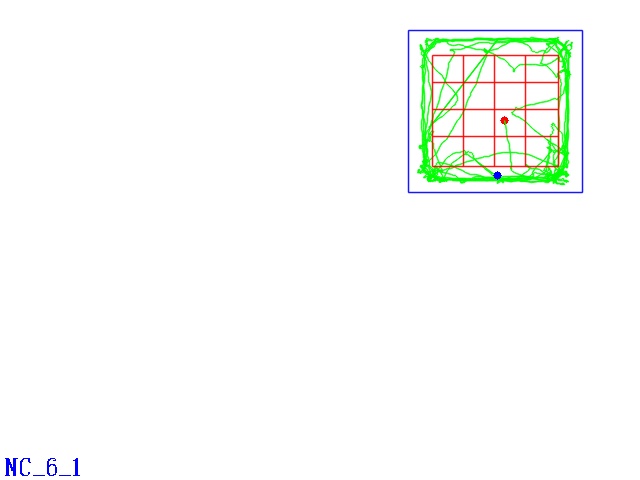

Supplement: S1 File — These materials provide additional support for the results presented in this study. (ZIP) [file pone.0346888.s005.zip › Open Field_Figure/Figure_Sham/S-6-1_20241008203633.jpg]

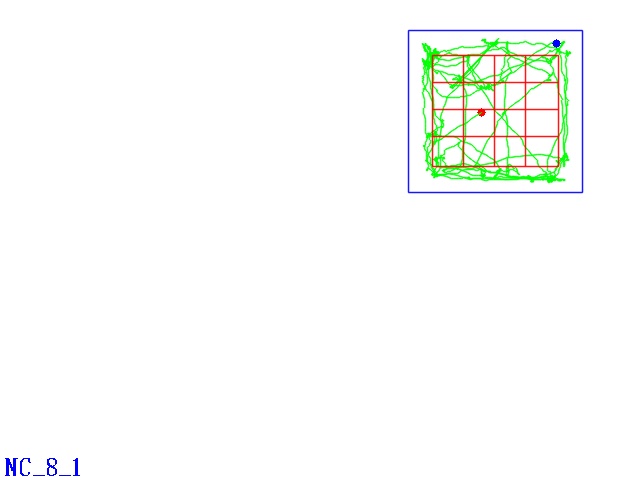

Supplement: S1 File — These materials provide additional support for the results presented in this study. (ZIP) [file pone.0346888.s005.zip › Open Field_Figure/Figure_Sham/S-8-1_20241008203633.jpg]
